# VGLL4 plays a critical role in heart valve development and homeostasis

**DOI:** 10.1371/journal.pgen.1007977

**Published:** 2019-02-21

**Authors:** Wei Yu, Xueyan Ma, Jinjin Xu, Andreas Wilhelm Heumüller, Zhaoliang Fei, Xue Feng, Xiaodong Wang, Kuo Liu, Jinhui Li, Guizhong Cui, Guangdun Peng, Hongbin Ji, Jinsong Li, Naihe Jing, Hai Song, Zhiqiang Lin, Yun Zhao, Zuoyun Wang, Bin Zhou, Lei Zhang

**Affiliations:** 1 State Key Laboratory of Cell Biology, CAS Center for Excellence in Molecular Cell Science, Innovation Center for Cell Signaling Network, Shanghai Institute of Biochemistry and Cell Biology, Chinese Academy of Sciences, University of Chinese Academy of Sciences, Shanghai, China; 2 Institute for Cardiovascular Regeneration, Goethe-University Hospital, Frankfurt, Germany; 3 School of Life Science and Technology, ShanghaiTech University, Shanghai, China; 4 Life Sciences Institute and Innovation Center for Cell Signaling Network, Zhejiang University, Hangzhou, China; 5 Masonic medical research institute, Utica, NY, United States of America; 6 The Collaborative Innovation Center for Cardiovascular Disease Translational Medicine, Nanjing Medical University, Nanjing, China; Indiana University Purdue University at Indianapolis, UNITED STATES

## Abstract

Heart valve disease is a major clinical problem worldwide. Cardiac valve development and homeostasis need to be precisely controlled. Hippo signaling is essential for organ development and tissue homeostasis, while its role in valve formation and morphology maintenance remains unknown. VGLL4 is a transcription cofactor in vertebrates and we found it was mainly expressed in valve interstitial cells at the post-EMT stage and was maintained till the adult stage. Tissue specific knockout of VGLL4 in different cell lineages revealed that only loss of VGLL4 in endothelial cell lineage led to valve malformation with expanded expression of YAP targets. We further semi-knockout YAP in VGLL4 ablated hearts, and found hyper proliferation of arterial valve interstitial cells was significantly constrained. These findings suggest that VGLL4 is important for valve development and manipulation of Hippo components would be a potential therapy for preventing the progression of congenital valve disease.

## Author summary

VGLL4, a new member of the Hippo pathway, is intensively investigated in inhibition of tumor progression via competing with YAP to bind TEADs, but its role in cardiovascular field remains unclear. Here we generated VGLL4 knockout mouse line and VGLL4-eGFP reporter mouse line. VGLL4-eGFP reporter mouse line showed VGLL4 was mainly expressed in valve interstitial cells from post-EMT stage to adult stage. Genetic loss of function and lineage tracing data demonstrated only endothelial loss of VGLL4 led to valve malformation with up-regulation of YAP targets. Of note, semi-knockout YAP could rescue this phenotype of VGLL4 knockouts. This is the first study to show the Hippo pathway plays a critical role in valve remodeling, maturation and homeostasis. Our findings suggest that mutations in VGLL4 may underlie human congenital heart valve dysplasia.

## Introduction

Heart valves are critical for orchestrating the unidirectional blood flow during the cardiac cycle, ensuring the correct and efficient function of the heart [1]. Heart valve disease has a high morbidity and mortality worldwide and their incidence increases with age [2]. Heart arterial valve development starts with endothelial-to-mesenchymal transition (EMT) to form the endocardial cushion, in which valvular endothelial cells (VEC) invade into the outflow tract (OFT) and trans-differentiate into valvar interstitial cells (VIC) to form the proximal cushions. Subsequently, neural crest cells migrate into the OFT to form the distal cushion. Then the valve primordia undergo extracellular matrix (ECM) remodeling and elongate into thin mature leaflets with three separate layers: the ventricularis (elastin-rich), fibrosa (fibrillar collagen-rich) and spongiosa (proteoglycan-rich). During this process, cell proliferation and apoptosis of VEC and VIC are precisely regulated, and, once disturbed, it will result in valve malformation [3, 4]. Valve disease can occur due to impaired cardiac cushion development, following infections or other elevated stress conditions such as heart attack, and usually lead to calcification or stenosis, eventually resulting in congestive heart failure [5]. However, the molecular mechanism underlying valve maturation and homeostasis remains incompletely understood so far.

The Hippo pathway is an evolutionarily conserved pathway from *Drosophila* to human which regulates cell proliferation, differentiation, stem cell fate determination and organ size in multiple organs and tissues [6]. Numerous studies demonstrate that the Hippo pathway is not only critical for embryonic cardiac development, perinatal cardiomyocyte proliferation and heart regeneration [7–9], but also plays important roles in multiple cardiovascular diseases, such as myocardial infarction, cardiac hypertrophy and atherosclerosis [10–12]. The Vestigial-like (VGLL) family includes its four members VGLL1-4, which mainly serve as transcription cofactors in vertebrates [13]. We have previously identified VGLL4 in mice and its homolog SdBP in *Drosophila* that competes with YAP for TEADs binding, which results in the inhibition of YAP-induced overgrowth and tumor genesis in gastric and lung cancer [14–16]. Interestingly, VGLL4 is the only member of the VGLL family expressed in the heart [13]. While the role of VGLL4 in heart valve development and homeostasis has remained unknown.

Here we constructed a VGLL4 reporter mouse line to investigate the VGLL4 expression pattern and found that the expression of VGLL4 increased dramatically in endothelial and interstitial cells in the valve region at the post-EMT stage and was maintained till the adult stage. We further used endothelial specific and neural crest specific Cre mice to knock out VGLL4 in different cell lineages that have been reported to contribute to valve interstitial cells. We found that only endothelial loss of VGLL4 led to valve malformation. Mechanistically, after semi-knockout of YAP, the phenotype of arterial valves in VGLL4 deleted heart was rescued, indicating that VGLL4 was required for normal valve development by antagonizing YAP activity. Together, we identified a key role of VGLL4 in valve development and maintenance.

## Results

### Constitutive knockout of VGLL4 leads to semilunar valve malformation

**Fig 1:**
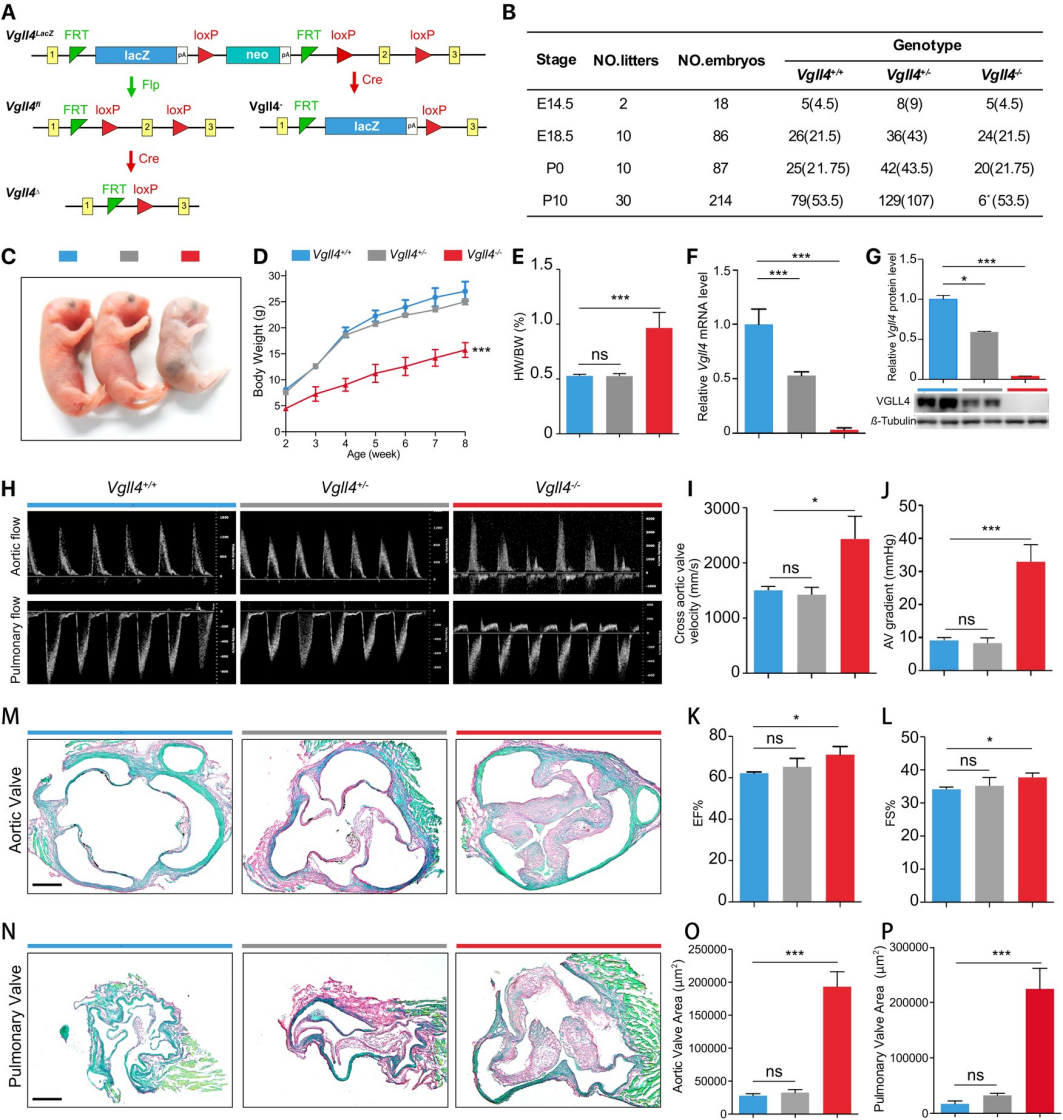
VGLL4 knockout mouse shows serious phenotype on valves. (A) Schematic representation of the knock out strategy of the Vgll4 knockout first (LacZ), Vgll4 flox (fl), Vgll4 minus (-) and Vgll4 delta (Δ) allel. (B) Table summarizes the postnatal lethal phenotype of *Vgll4*^*-/-*^ mice (expected Mendelian values in brackets). (C) Gross view shows that P1 *Vgll4*^*-/-*^ mice are underdeveloped compared with *Vgll4*^*+/-*^ and *Vgll4*^*+/+*^ controls. (D) Body weight trace from 2 to 8 weeks old of age of *Vgll4*^*-/-*^ (n = 5), *Vgll4*^*+/-*^ (n = 10) and *Vgll4*^*+/+*^ (n = 8) mice. (E) The ratio of heart weight to body weight (HW/BW) is increased in *Vgll4*^*-/-*^ mice (n = 5) compared with *Vgll4*^*+/-*^ (n = 10) and *Vgll4*^*+/+*^ (n = 8) mice at 8 weeks of age. (F) Quantitative PCR of neonatal heart total RNA indicates Vgll4 knockout efficiency in *Vgll4*^*-/-*^ and control hearts (n = 4). (G) Western Blot of neonatal heart total protein shows VGLL4 protein level on *Vgll4*^*-/-*^ and *Vgll4*^*+/-*^ and *Vgll4*^*+/+*^ controls and quantitation (n = 2). (H) Pulse-wave Doppler analysis from the aorta and pulmonary artery of 8-week-old *Vgll4*^*-/-*^ mice and *Vgll4*^*+/-*^, *Vgll4*^*+/+*^ littermate controls, demonstrates *Vgll4*^*-/-*^ heart have aortic and pulmonary regurgitation. (I,J) Doppler analysis shows that 8-week-old *Vgll4*^*-/-*^ mice (n = 3) have significantly increased cross aortic valves systolic velocity (I), and pressure gradient (J) compared with the *Vgll4*^*+/-*^ (n = 4) and *Vgll4*^*+/+*^ (n = 5) controls. (K,L) Echocardiography shows that 8-week-old *Vgll4*^*-/-*^ mice have slightly increased ejection fraction (K) and fractional shortening (L) compared with age-matched controls (n = 5). (M) Sirius Red staining shows thickened aortic valve in *Vgll4*^*-/-*^ group in 8-week-old of age compared with age-matched controls. (N) Sirius Red staining shows thicken pulmonary valve in *Vgll4*^*-/-*^ group in 8-week-old of age compared with age-matched controls. (O) Aortic valve area per section was measured (n = 4). (P) Pulmonary valve area per section was measured (n = 4). EF: ejection fraction. FS: fractional shortening. Scale bar = 200μm. *P<0.05, **P<0.01, ***P<0.005, ns: no significance.

To study the function of VGLL4 during heart valve development, we first generated a *Vgll4* targeted allele by knockout-first strategy [17] (Figs 1A and S1A). By directly crossing *Vgll4*^*lacZ*^ with female *Sox2*^*Cre+*^ mice, which expresses Cre recombinase in all cells of the embryo during the blastocyst stage of development, we obtained viable heterozygous *Vgll4*^*+/-*^ at an expected Mendelian ratio. *Vgll4*^*+/-*^ mice were then intercrossed to generate *Vgll4*^*-/-*^ knockout mutants. Besides, we got conditional alleles (*Vgll4*^*fl*^) with two loxP-sites on either side of the second exon and restored gene activity after removal of the gene-trap cassette by FLP recombinase (Fig 1A). By subsequent crossing with tissue specific Cre mouse line, we could further get conditional deleted *Vgll4Δ* allele (Fig 1A). We collected embryos at different stages and noticed a significant reduction of *Vgll4*^*-/-*^ mice from P1 to later stages, suggesting an overall mortality shortly after birth (Figs 1B and S2). Although only few *Vgll4*^*-/-*^ mice survived over 8 weeks, the body size of these mice was significantly smaller than that of the *Vgll4*^*+/-*^ and *Vgll4*^*+/+*^ littermate controls from neonatal to adult stage (Fig 1C and 1D). We also found that all organs, except heart, were smaller than *Vgll4*^*+/-*^ and *Vgll4*^*+/+*^ controls, this is reflected by the ratio of heart weight to body weight which is significantly increased in *Vgll4*^*-/-*^ mice (Fig 1E). Thus we focus our research on the heart.

We first confirmed the endogenous VGLL4 mRNA and protein expression by qRT-PCR and western blot that VGLL4 was efficiently ablated in *Vgll4*^*-/-*^ hearts compared with that of littermate *Vgll4*^*+/-*^ and *Vgll4*^*+/+*^ controls (Fig 1F and 1G). Echocardiography was performed in 8 weeks *Vgll4*^*-/-*^ mice and littermate controls, as all the *Vgll4*^*-/-*^ mice died in 14 weeks (S2 Fig). Pulse-wave Doppler analysis indicated regurgitation across the aortic valve (AoV) and the pulmonary valve (PV) in *Vgll4*^*-/-*^ hearts, whereas *Vgll4*^*+/-*^ and *Vgll4*^*+/+*^ hearts showed normal pulse-wave Doppler images (Fig 1H). Doppler analysis also revealed a significant increase of blood velocity and pressure gradient across the aortic valves in *Vgll4*^*-/-*^ hearts compared with *Vgll4*^*+/-*^ and *Vgll4*^*+/+*^ littermate controls (Fig 1I and 1J). Besides, ejection fraction (EF), fractional shortening (FS) were increased in *Vgll4*^*-/-*^ hearts (Fig 1K and 1L and S1 Table), suggesting that *Vgll4*^*-/-*^ hearts developed aortic stenosis and undergo compensatory hypertrophy, a feature of hypercontractile state. Sirius Red staining of cross sections in AoV and PV showed that all leaflets were thickened in *Vgll4*^*-/-*^ mice at both the adult and neonatal stages (Figs 1M–1P and S3). In contrast to AoV and PV, the mitral and tricuspid valves appeared normal at neonatal stage (S3 Fig). Besides, we detected a dramatic increase of proliferation of VIC at E15.5 heart valves, while nuclear density was normal compared with *Vgll4*^*+/+*^ controls (S4 Fig), suggesting that loss of VGLL4 leads to VIC over proliferation and malformation of arterial valves.

### VGLL4 is expressed in heart valves post-EMT

**Fig 2:**
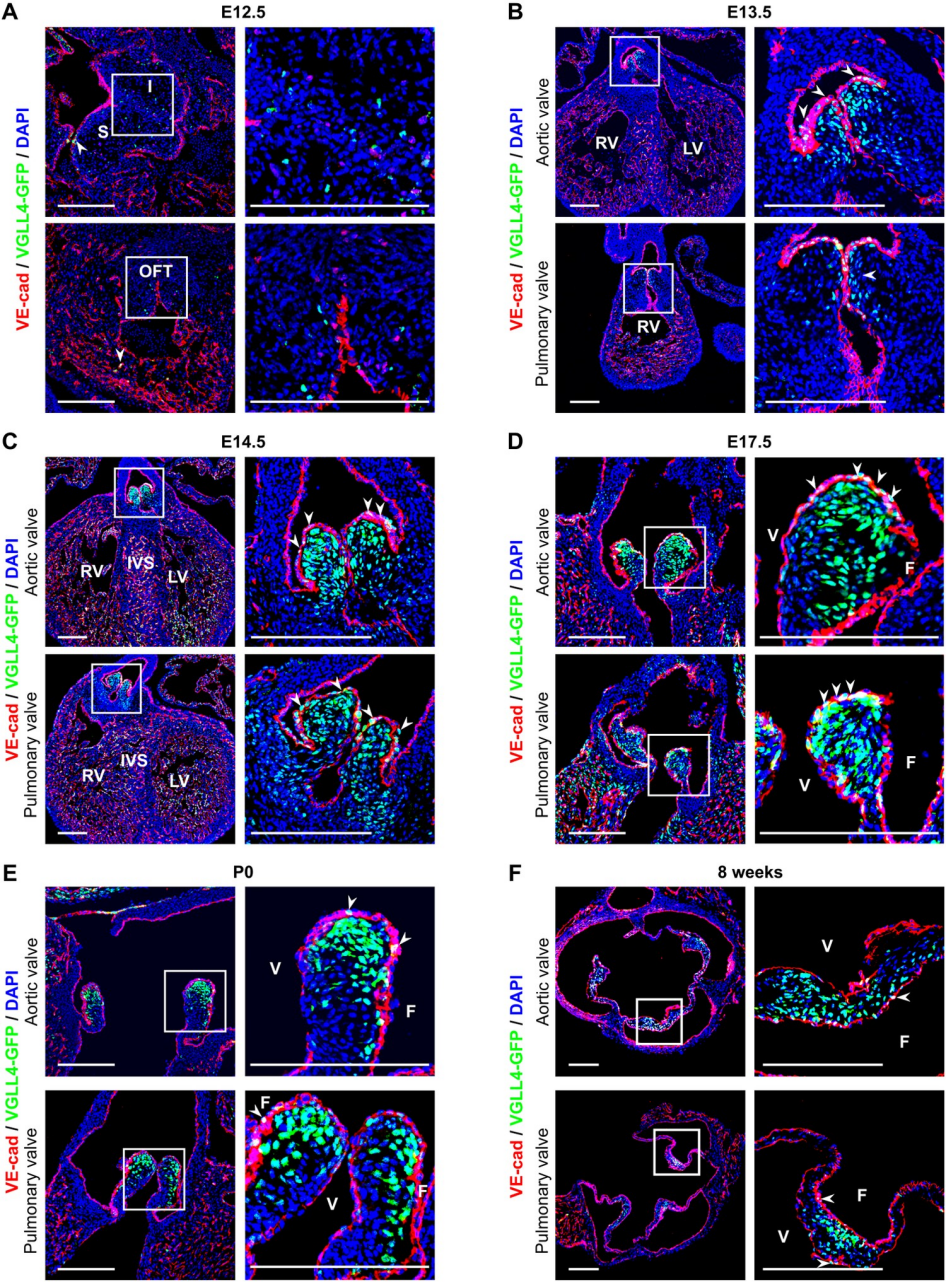
The spatiotemporal patterns of VGLL4-GFP fusion protein expression. (A-F) Heart sections from E12.5, E13.5, E14.5, E17.5, P0 and 8 weeks were performed immunofluorescence staining with GFP (Green), endothelial maker VE-cadherin (red) and DAPI (blue). (A) VGLL4-GFP fusion protein is seldom detected in cushion mesenchyme and endothelial cells at E12.5 (white arrows). (B) VGLL4-GFP begins to be expressed in the aortic valve (AoV) and pulmonary valves (PV) at E13.5. (C) VGLL4-GFP is intensively expressed in VICs and VECs on the valve region and ventricular endothelial cells at E14.5. (D) VGLL4-GFP expression remains intensively in the valve region, and GFP signal is decreased in the ventricularis side in the aortic valve at E17.5. (E) VGLL4-GFP expression is predominately found in VICs, while in VEC and ventricularis side, GFP signal is decreased in both aortic valves and pulmonary valves at neonatal stage. (F) At adult stage (8-week-old), VGLL4-GFP expression remains intensively in valve ventricularis side and seldom detected in VECs (white arrows). I, inferior cushion; S, superior cushion; OFT, outflow tract; LV, left ventricle; RV, right ventricle; IVS, intraventricular septum; V, ventricularis; F, fibrosa; Scale Bar = 200μm.

In order to investigate the expression pattern of VGLL4 in heart valves precisely, we generated a *Vgll4*^*Vgll4-eGFP/+*^ reporter mouse line in which VGLL4-eGFP fusion protein expression is under the control of the endogenous VGLL4 promoter, and GFP staining therefore reflects VGLL4 expression pattern (S5 Fig). We intercrossed heterozygous *Vgll4*^*Vgll4-eGFP/+*^ mice and collected homozygous heart tissues at different development stages and found that the GFP signal was rarely detected in the cardiac cushion at embryonic day 12.5 (E12.5) and began to be expressed on VEC and VIC in the tip of the AoV and PV at E13.5 when EMT was finished, and OFT was separated and valve primordium underwent ECM remodeling (Fig 2A and 2B). VGLL4-eGFP was robustly expressed in the whole valve region and endothelial cells of the myocardium at E14.5 (Fig 2C). At E17.5, GFP signal was widely detected in other cell types in addition to endothelial cells (Fig 2D). Our data also showed that GFP signals were more inclined to be distributed in the fibrosa side throughout the perinatal (E17.5) to adult stage (8 weeks) (Fig 2D–2F). We therefore propose that VGLL4 plays a role in valve remodeling soon after heart cushion formed via EMT.

### The function of VGLL4 in valves is cell lineage dependent

**Fig 3:**
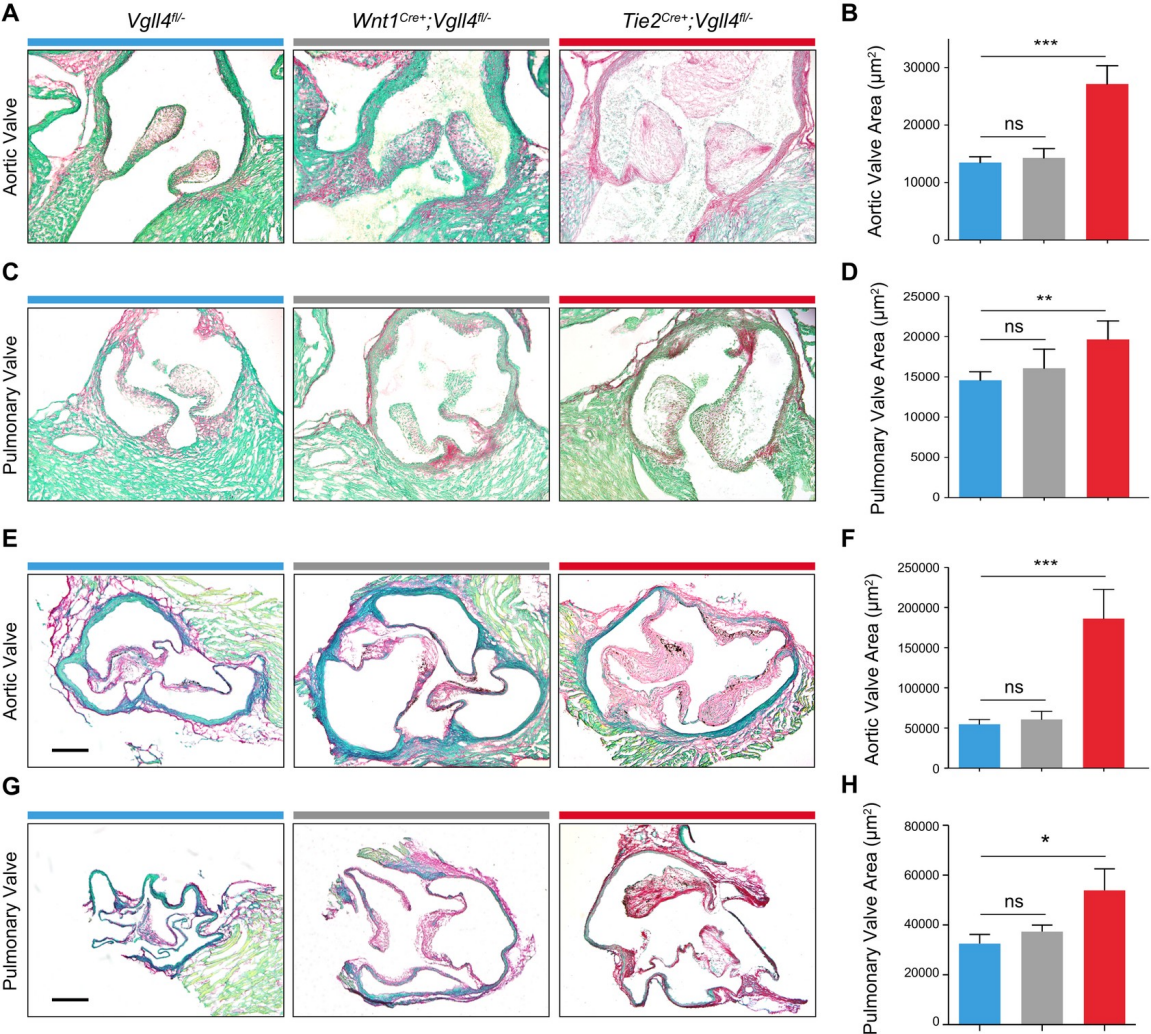
VGLL4 ablation in endothelial cells leads to arterial valves thickening. (A,C) Sirius Red staining of postnatal (P0) arterial heart valves from *Wnt1*^*Cre+*^*;Vgll4*^*fl/-*^; *Tie2*^*Cre+*^*;Vgll4*^*fl/-*^ and *Vgll4*^*fl/-*^ controls. (B,D) Quantitation of aortic valve area (B) and pulmonary valve area (D) from postnatal *Wnt1*^*Cre+*^*;Vgll4*^*fl/-*^ (n = 4), *Tie2*^*Cre+*^*;Vgll4*^*fl/-*^ (n = 7) and *Vgll4*^*fl/-*^ controls (n = 11). (E,G) Sirius Red staining of adult (36 weeks) arterial valves from *Wnt1*^*Cre+-*^*;Vgll4*^*fl/-*^; *Tie2*^*Cre+*^*;Vgll4*^*fl/-*^ and *Vgll4*^*fl/-*^ controls. (F,H) Quantitation of aortic valve area (F) and pulmonary valve area (H) from adult *Wnt1*^*Cre+*^*;Vgll4*^*fl/-*^ (n = 5), *Tie2*^*Cre+*^*;Vgll4*^*fl/-*^ (n = 5) and *Vgll4*^*fl/-*^ controls (n = 8). Scale bar = 200μm. *P<0.05, **P<0.01, ns: no significance.

Previous studies indicated that endothelial and neural crest cells are the two main sources of semilunar valves[18]^,^[19]. We first used *Rosa*^*fsRFP/+*^ reporter mice crossed with either *Tie2*^*Cre+*^ or *Wnt1* ^*Cre+*^ transgenic mouse line and confirmed the endothelial cells or neural crest cells were involved in neonatal heart valve development (S6 Fig). To additionally evaluate the function of VGLL4 in the different lineages, we crossed *Tie2* ^*Cre+*^ and *Wnt1*^*Cre+*^ mice with *Vgll4*^*fl/-*^ mice to inactivated VGLL4 in the respective cell lineages. All the mice survived normally and the pups were obtained with expected Mendelian ratio (22/88 for *Tie2* ^*Cre+*^*;Vgll4*^*fl/-*^, 23/88 for *Vgll4*^*fl/-*^, 23/88 for *Tie2* ^*Cre+*^*;Vgll4*^*fl/fl*^, 20/88 for *Vgll4*^*fl/fl*^). Histologic data showed that only endothelial loss of VGLL4 (*Tie2* ^*Cre+*^*;Vgll4*^*fl/-*^) led to both aortic and pulmonary valves thickening in neonatal hearts (P0) (Fig 3A–3D). Abnormal arterial valve morphology could also be detected at adult stage (9-month-old) in *Tie2*^*Cre+*^*;Vgll4*^*fl/-*^ heart (Fig 3E–3H), whereas loss of VGLL4 in the neural crest lineage (*Wnt1*^*Cre+*^*;Vgll4*^*fl/-*^) didn’t lead to valve malformation in neither the neonatal nor the adult stage (Fig 3). These data demonstrate that endothelial VGLL4 is essential for arterial valve development.

### Loss of VGLL4 in valve endothelial cells results in left ventricular hypertrophy

**Fig 4:**
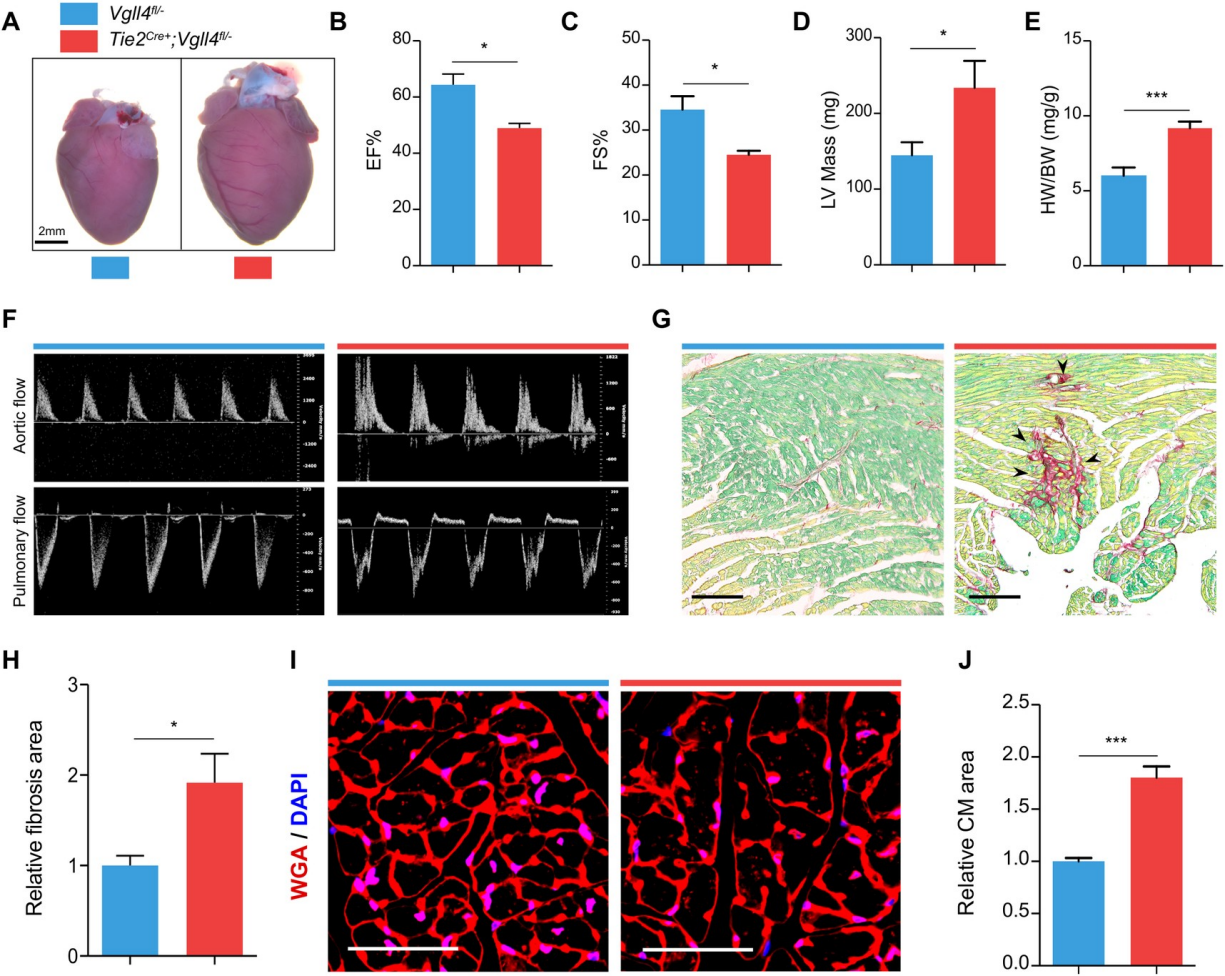
Knock out VGLL4 in valvar endothelial cells leads to hypertrophy and compromised heart function on later stage. (A) Gross front view of hearts from 9-month-old *Tie2*^*Cre+*^*;Vgll4*^*fl/-*^ and *Vgll4*^*fl/-*^ control hearts. Echocardiography shows that *Tie2*^*Cre+*^*;Vgll4*^*fl/-*^ (n = 4) mice have significantly decreased ejection fraction (B) and fractional shortening (C), and increased left ventricular mass (D) compared with age-matched *Vgll4*^*fl/-*^ (n = 6) control hearts. (E) *Tie2*^*Cre+*^*;Vgll4*^*fl/-*^ mice have significantly increased ratio of heart/body weight compared with age-matched *Vgll4*^*fl/-*^ controls. (F) Pulse-wave Doppler analysis from the aorta and pulmonary artery of 9-month-old *Tie2*^*Cre+*^*;Vgll4*^*fl/-*^ (n = 4) and *Vgll4*^*fl/-*^ (n = 10) control hearts, demonstrates *Tie2*^*Cre+*^*;Vgll4*^*fl/-*^ hearts have aortic regurgitation. (G) Sirius staining shows fibrosis area (black arrow indicated) detected in *Tie2*^*Cre+*^*;Vgll4*^*fl/-*^ heart sections. (H) Quantification of fibrosis area on *Tie2*^*Cre+*^*;Vgll4*^*fl/-*^ (n = 4) and control (n = 4) heart sections. (I) Immunostaining for WGA (Red) on heart sections shows cardiomyocyte cross-sectional borderline, DAPI is in blue. (J) The cross-sectional area of cardiomyocytes in *Tie2*^*Cre+*^*;Vgll4*^*fl/-*^ (n = 4) was measured and compared with *Vgll4*^*fl/-*^ control hearts (n = 4). EF: ejection fraction; FS: fractional shortening; LV: left ventricle; HW: heart weight; BW: body weight; CM: Cardiomyocyte. Scale Bar: black = 100μm; white = 50μm.*P<0.05,**P<0.01.

Although endothelial-specific VGLL4 knockout mice (*Tie2*^*Cre+*^*;Vgll4*^*fl/-*^) could survive, these mice developed severe cardiac hypertrophy at 36 weeks of age (Fig 4A). Echocardiography also showed that *Tie2*^*Cre+*^*;Vgll4*^*fl/-*^ hearts had decreased ejection fraction, fraction shortening (Fig 4B and 4C) and increased left ventricular mass (Fig 4D and S2 Table). These phenotypes were accompanied by an increase in the heart weight to body weight (HW/BW) ratio (Fig 4E). Additionally, Doppler analysis showed aortic and pulmonary regurgitation in *Tie2*^*Cre+*^*;Vgll4*^*fl/-*^ mice (Fig 4F). Sirius Red staining showed increased fibrosis in *Tie2*^*Cre+*^*;Vgll4*^*fl/-*^ heart sections (Fig 4G and 4H). WGA staining showed that the cross sectional area of cardiomyocyte was increased in *Tie2*^*Cre+*^*;Vgll4*^*fl/-*^ mice (Fig 4I and 4J), indicating cardiomyocyte hypertrophy. In addition, we detected capillary vessel distribution and endocardium in neonatal of cardiac chamber and found no obvious difference between *Tie2*^*Cre+*^*;Vgll4*^*fl/-*^ heart and *Vgll4*^*fl/-*^controls (S7 Fig), so we infer that the myocardial defect is due to secondary to primary valve malformation rather than endocardium Vgll4 ablation.

### VGLL4 deletion leads to increased endothelial-derived VIC proliferation

**Fig 5:**
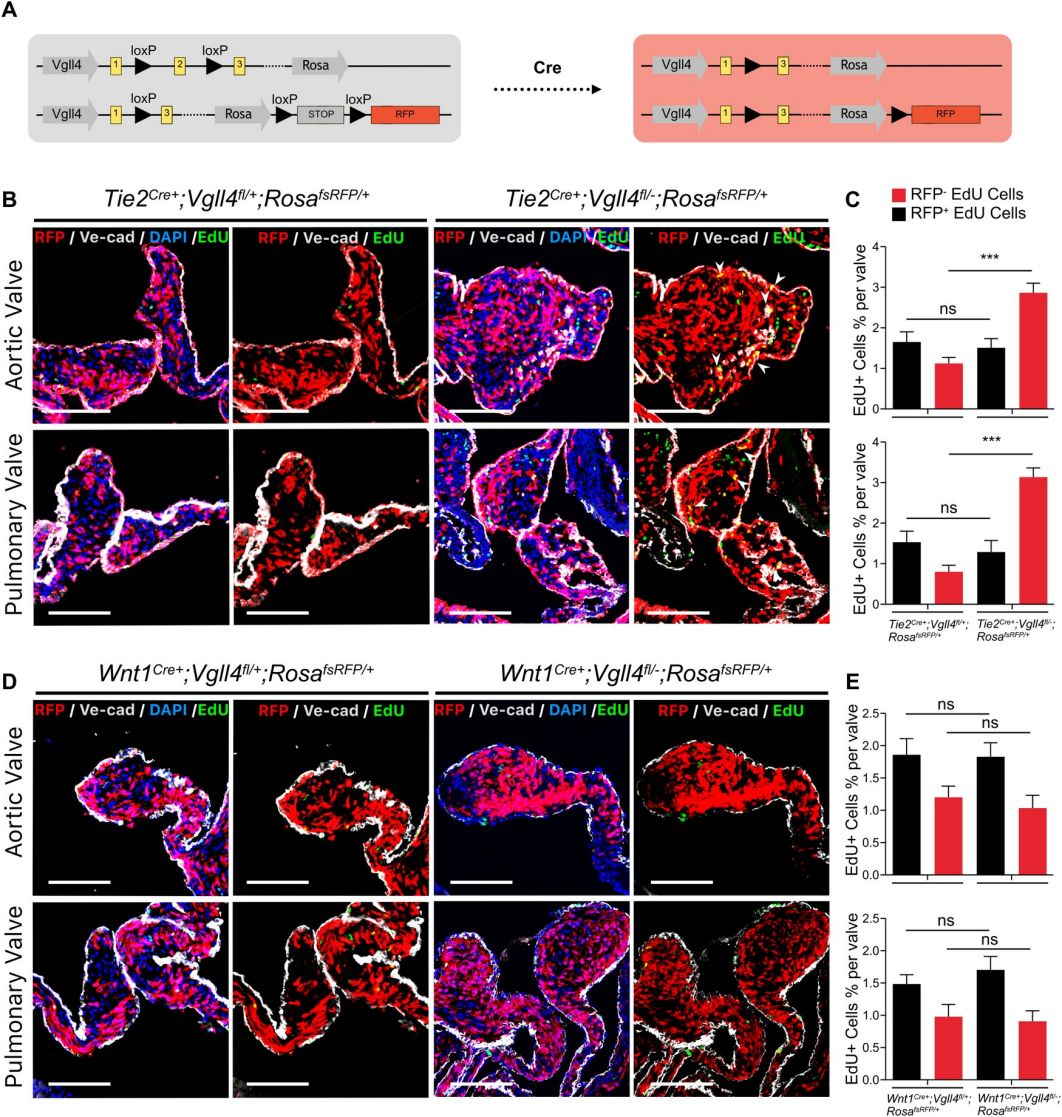
Cell proliferation is significantly increased from endothelial lineage loss of VGLL4 valves. (A) Schematic view of recombined Vgll4 allele and experimental strategy were used to create valves composed of VGLL4 semi-knockout (RFP-) and VGLL4 total knockout (RFP+) VIC populations. (B) Immunoflorecence analyses of RFP positive proliferating cells on P10 heart arterial valves from *Tie2*^*Cre+*^*;Vgll4*^*fl/-*^*;Rosa*^*fsRFP/+*^ (n = 5) and *Tie2*^*Cre+*^*;Vgll4*^*fl/+*^*;Rosa*^*fsRFP/+*^ controls (n = 5). DAPI is in blue, EdU is in green, VE-cad is in gray. (C) Quantification of the percentage from RFP^-^,EdU^+^ and RFP^+^,EdU^+^ cells per aortic or pulmonary valve. (D) Immunoflorecence analyses of RFP positive proliferating cells on P10 heart arterial valves from *Wnt*^*Cre+*^*;Vgll4*^*fl/-*^*;Rosa*^*fsRFP/+*^ (n = 5) and *Wnt*^*Cre+*^*;Vgll4*^*fl/+*^*;Rosa*^*fsRFP/+*^ (n = 5) controls. DAPI is in blue, EdU is in green, VE-cad is in gray. (E) Quantification of the percentage from RFP^-^,EdU^+^ and RFP^+^,EdU^+^ cells per aortic or pulmonary valve. AoV: aortic valve; PV: pulmonary valve. White arrows point proliferating endothelial lineage cells. Scale Bar = 100μm.***P<0.005, ns: no significance.

Given that no phenotype was observed on neural crest lineage (*Wnt1*^*Cre+*^*;Vgll4*^*fl/-*^), we hypothesized that VGLL4 mainly functioned in endothelial lineage VIC. To further strengthen this, we performed lineage tracing on the VGLL4 total knockout cell population. Fortunately, we obtained an allele that included both *Vgll4-minus* and *Rosa-fsRfp* at a very low probability as *Vgll4* and *Rosa* locus are in the same chromosome (Fig 5A). After crossing both *Tie2*^*Cre+*^*;Vgll4*^*fl/fl*^ and *Wnt1*^*Cre+*^*;Vgll4*^*fl/fl*^ with the recombined *Vgll4*^*fl/-*^*;Rosa*^*fsRFP/+*^ mice, we got *Tie2*^*Cre+*^*;Vgll4*^*fl/-*^*;Rosa*^*fsRFP/+*^ and *Wnt1*^*Cre+*^*;Vgll4*^*fl/-*^*;Rosa*^*fsRFP/+*^ mice, in which VGLL4 total knockout VIC were labeled with RFP and semi-knockout VIC were RFP negative. We collected hearts that paulse chased with EdU at P10 when the proliferation of VIC was almost finished in *Tie2*^*Cre+*^*;Vgll4*^*fl/+*^*;Rosa*^*fsRFP/+*^ control valves, we found numerous proliferating VICs were remain detectable in *Tie2*^*Cre+*^*;Vgll4*^*fl/-*^*;Rosa*^*fsRFP/+*^ valves (Fig 5B and 5C). Immunofluorescence staining unequivocally presented that robustly proliferating RFP positive interstitial cells in *Tie2*^*Cre+*^*;Vgll4*^*fl/-*^*;Rosa*^*fsRFP/+*^ mice, indicating their endothelial origin, both in the AoV and PV. In contrast, we could hardly detect proliferating RFP positive cells in *Tie2*^*Cre+*^*;Vgll4*^*fl/+*^*;Rosa*^*fsRFP/+*^ controls or *Wnt1*^*Cre+*^*;Vgll4*^*fl/-*^*;Rosa*^*fsRFP/+*^ and *Wnt1*^*Cre+*^*;Vgll4*^*fl/+*^*;Rosa*^*fsRFP/+*^ semilunar valves (Fig 5B to Fig 5E). In addition, we also found that the percentage of EdU positive and RFP negative VICs in *Tie2*^*Cre+*^*;Vgll4*^*fl/-*^*;Rosa*^*fsRFP/+*^ have no significant increase compared with *Tie2*^*Cre+*^*;Vgll4*^*fl/+*^*;Rosa*^*fsRFP/+*^ controls, this means VGLL4 palys its role in endothelial derived VICs and have no non-cell autonomous effect in other RFP negative VICs (Fig 5C). In conclusion, these data implicate that VGLL4 knockout in endothelial cells results in an enhanced proliferation of endothelial-derived VICs, ultimately causing valve thickening.

We also detected ECM distribution on adult semilunar valves by perform immunostaining of ECM markers: Periostin (Postn), CollagenIII (ColIII) and Versican (Vcan), we found that ColIII and Vcan distribution is incoherent compared with *Vgll4*^*fl/-*^ controls (S8 Fig). At the same time, we analyzed DAPI number of adult semilunar valves, and found the number of cell nuclear per valve was increased in *Tie2*^*Cre+*^*;Vgll4*^*fl/-*^ valves but the ratio of DAPI to valves area have no significant difference between *Tie2*^*Cre+*^*;Vgll4*^*fl/-*^ heart and *Vgll4*^*fl/-*^controls (S8 Fig). These data demonstrate that the malformation of *Tie2*^*Cre+*^*;Vgll4*^*fl/-*^ adult valves comes from prolonged cell proliferation, valves contain more cells rather than replace by excess ECM deposition.

### Semi-knockout of YAP protects VGLL4 depleted semilunar valves from malformation

**Fig 6:**
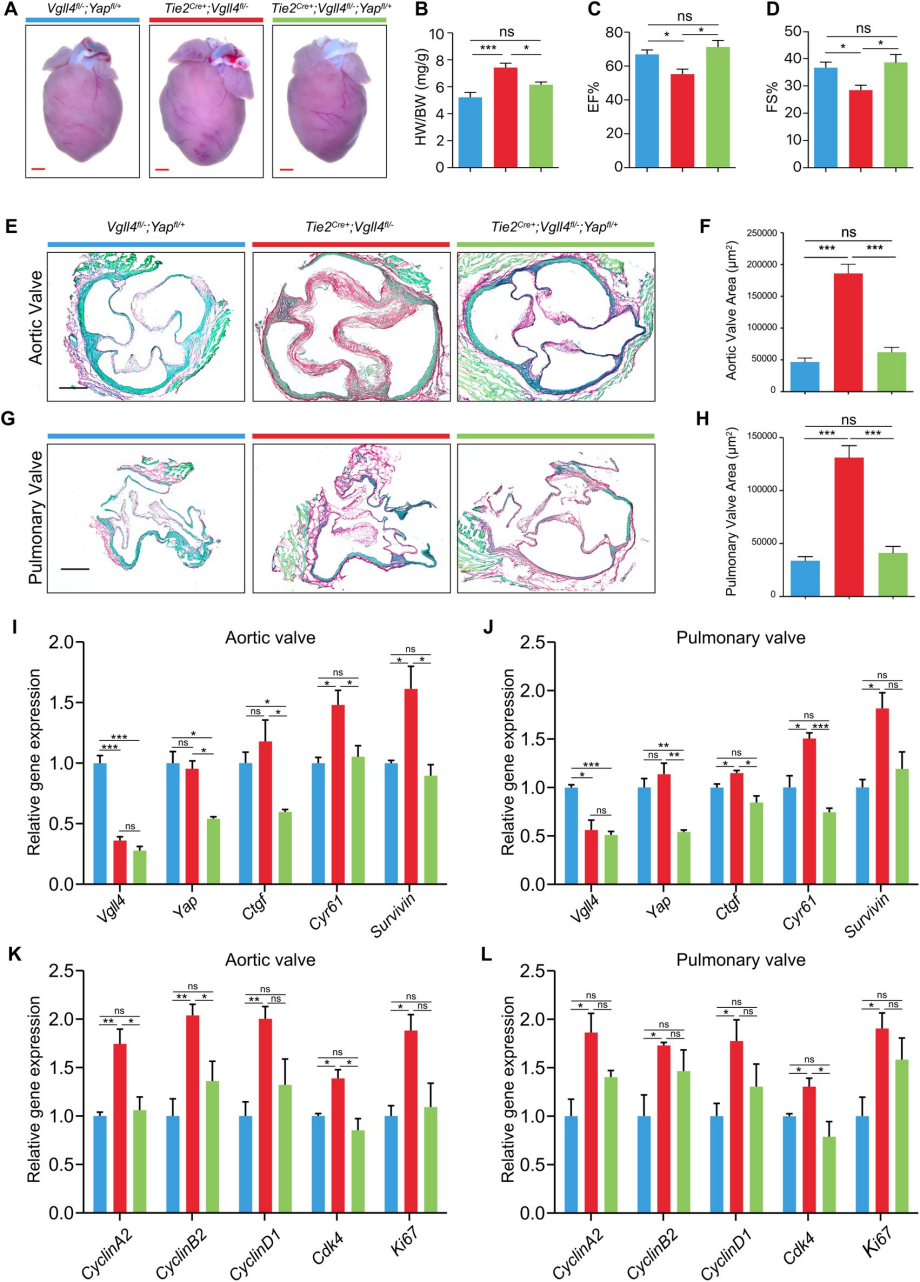
Semi-knockout YAP rescue heart function from valve thickening. (A) Gross front view of hearts from 9-month-old *Tie2*^*Cre+*^*;Vgll4*^*fl/-*^; *Tie2*^*Cre+*^*;Vgll4*^*fl/-*^*;Yap*^*fl/+*^ and *Vgll4*^*fl/-*^*;Yap*^*fl/+*^ hearts. (B) The heart weight/body weight (HW/BW) ratio of *Tie2*^*Cre+*^*;Vgll4*^*fl/-*^; *Tie2*^*Cre+*^*;Vgll4*^*fl/-*^*;Yap*^*fl/+*^ and *Vgll4*^*fl/-*^*;Yap*^*fl/+*^ hearts at 9-month-old age (n = 4). (C,D) Echocardiographic parameters of ejection fraction (C) and fractional shortening (D) are decreased in *Tie2*^*Cre+*^*;Vgll4*^*fl/-*^ (n = 6) hearts, but did not change significantly in the *Tie2*^*Cre+*^*;Vgll4*^*fl/-*^*;Yap*^*fl/+*^ (n = 5) hearts compared with *Vgll4*^*fl/-*^*;Yap*^*fl/+*^ (n = 7) hearts. (E,G) Representative Sirius Red staining of adult arterial heart valves from *Vgll4*^*fl/-*^*;Yap*^*fl/+*^; *Tie2*^*Cre+*^*;Vgll4*^*fl/-*^ and *Tie2*^*Cre+*^*;Vgll4*^*fl/-*^*;Yap*^*fl/+*^ mice. (F,H) Quantitation of aortic valve area (F) and pulmonary valve area (H) from adult *Vgll4*^*fl/-*^*;Yap*^*fl/+*^; *Tie2*^*Cre+*^*;Vgll4*^*fl/-*^; *Tie2*^*Cre+*^*;Vgll4*^*fl/-*^*;Yap*^*fl/+*^ mice (n = 5). (I,J) *Vgll4*, *Yap* and Hippo downstream targets (*Ctgf*, *Cyr61* and *Survivin*) expression was measured by qRT-PCR in 2-month-old *Vgll4*^*fl/fl*^*;Yap*^*fl/+*^; *Tie2*^*Cre+*^*;Vgll4*^*fl/fl*^ and *Tie2*^*Cre+*^*;Vgll4*^*fl/fl*^*;Yap*^*fl/+*^ mice AoV (I) and PV (J) results were normalized to *Gapdh* and compared to *Vgll4*^*fl/fl*^*;Yap*^*fl/+*^ Controls (n = 3). (K,L) Proliferation makers (*Cyclin A2*, *CyclinD1*, *CDK4* and *Ki67*) expression was measured by qPCR in 2-month-old *Vgll4*^*fl/fl*^*;Yap*^*fl/+*^; *Tie2*^*Cre+*^*;Vgll4*^*fl/fl*^ and *Tie2*^*Cre+*^*;Vgll4*^*fl/fl*^*;Yap*^*fl/+*^ mice AoV (K) and PV (L), results then normalized to *Gapdh* and compared to *Vgll4*^*fl/fl*^*;Yap*^*fl/+*^ controls (n = 3). Scale bar = 200μm. *P<0.05, **P<0.01, ***P<0.005, ns: no significance.

Given the competition of YAP and VGLL4 for the binding to TEADs, and the requirement of YAP for normal proliferation during development, we hypothesized that VGLL4 regulated VIC proliferation during valve remodeling via suppression of the function of YAP. We first detected YAP is widely expressed in E15.5 hearts, but its localization have no significant difference between *Tie2*^*Cre+*^*;Vgll4*^*fl/-*^ and *Vgll4*^*fl/-*^ controls (S9 Fig). Then we further crossed *Vgll4*^*fl/fl*^*;Yap*^*fl/fl*^ mice with *Tie2*^*Cre+*^*;Vgll4*^*fl/-*^ mice and generated *Tie2*^*Cre+*^*;Vgll4*^*fl/-*^*;Yap*^*fl/+*^ mice to knock out half of the YAP on endothelial cells in VGLL4 deleted hearts. We found *Tie2*^*Cre+*^*;Vgll4*^*fl/-*^*;Yap*^*fl/+*^ hearts were normal compared with *Vgll4*^*fl/-*^*;Yap*^*fl/+*^ controls, but *Tie2*^*Cre+*^*;Vgll4*^*fl/-*^ mice developed pathological hypertrophic response, which was further verified by the ratio of HW/BW (Fig 6A and 6B). Echocardiography also showed that YAP semi-knockouted hearts shows normal cardiac contractility while *Tie2*^*Cre+*^*;Vgll4*^*fl/-*^ hearts had decreased ejection fraction, fraction shortening compared with *Vgll4*^*fl/-*^*;Yap*^*fl/+*^ controls (Fig 6C and 6D and S3 Table). Besides, the AoV and PV were normal in *Tie2*^*Cre+*^*;Vgll4*^*fl/-*^*;Yap*^*fl/+*^ mice compared with those of *Tie2*^*Cre+*^*;Vgll4*^*fl/-*^ and *Vgll4*^*fl/-*^*;Yap*^*fl/+*^ littermate hearts (Fig 6E–6H). Furthermore, qRT-PCR of adult valves of *Tie2*^*Cre+*^*;Vgll4*^*fl/fl*^ and *Tie2*^*Cre+*^*;Vgll4*^*fl/fl*^*;Yap*^*fl/+*^, *Vgll4*^*fl/fl*^*;Yap*^*fl/+*^ controls, data confirmed strong down-regulation of Vgll4 transcripts, besides, YAP targets *Ctgf*, *Cyr61* and *Survivin* were up-regulated in *Tie2*^*Cre+*^*;Vgll4*^*fl/fl*^ aortic and pulmonary valves, but this up-regulation was not detectable with additional YAP semi-knockout valves (Fig 6I and 6J). We also analyzed expression of proliferation marker genes (*CyclinA2*, *CyclinB2*, *CyclinD1*, *Cdk4*, *Ki67*) and found that endothelial loss of VGLL4 resulted in up-regulation of the cell cycle associated genes, while semi-knockout YAP rescued these changes (Fig 6K and 6L). Collectively, the data indicates semi-knockout of YAP could rescue heart valve malformation induced by VGLL4 deletion.

### VGLL4-YAP balances the proliferation and apoptosis of valve interstitial cells

**Fig 7:**
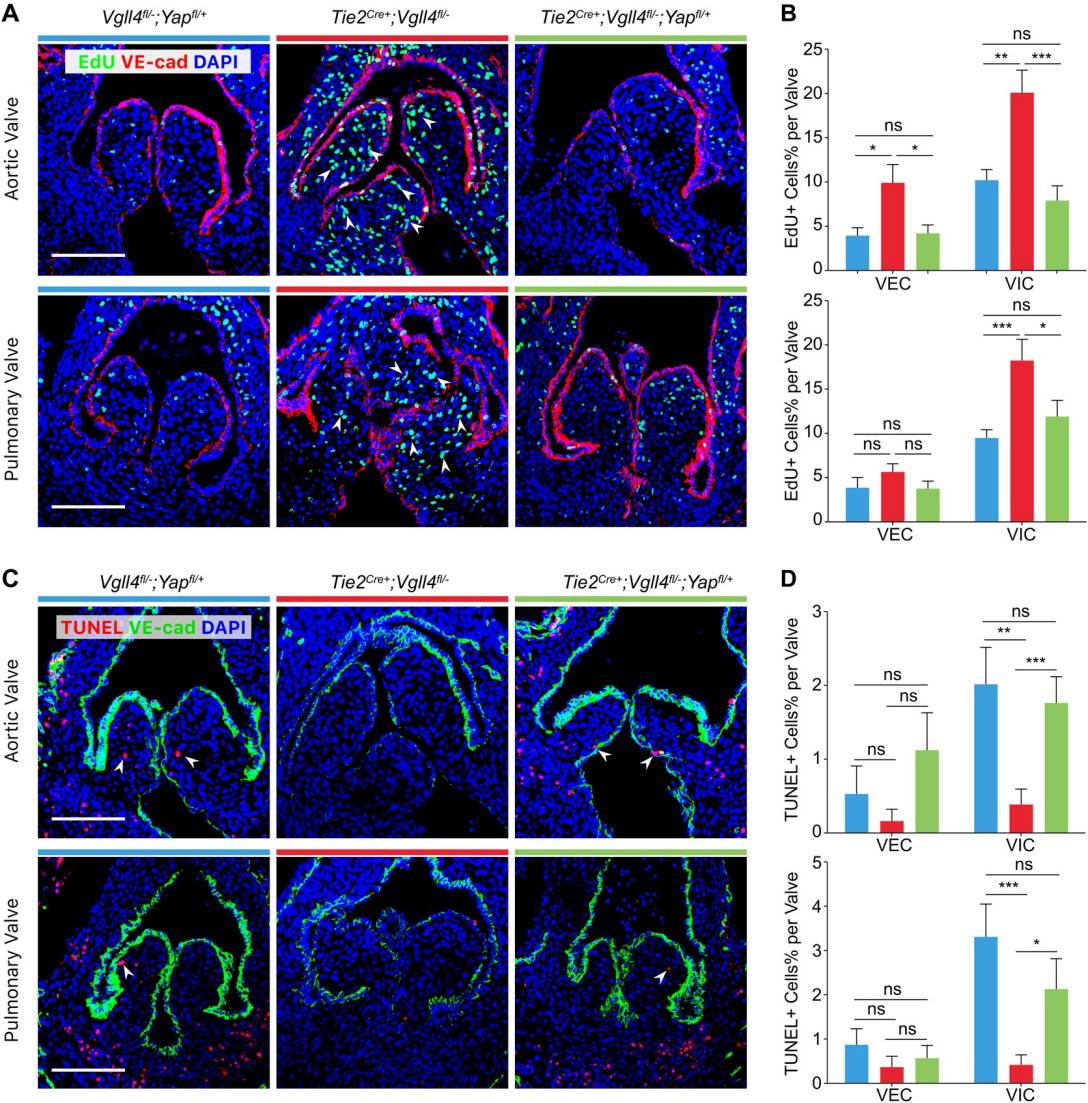
VGLL4 negatively regulates proliferation and promotes apoptosis of VICs during development of arterial valves. (A) EdU staining shows proliferating cells (green) in arterial valves of E15.5 *Vgll4*^*fl/-*^*;Yap*^*fl/+*^; *Tie2*^*Cre+*^*;Vgll4*^*fl/-*^ and *Tie2*^*Cre+*^*;Vgll4*^*fl/-*^*;Yap*^*fl/+*^ embryos. VE-cad staining marks VECs (red). (B) Quantitative results of EdU+ cells of VECs and VICs in each individual leaflet (n = 5). (C) TUNEL assay shows apoptotic cells (red) in arterial valves of E15.5 *Vgll4*^*fl/-*^*;Yap*^*fl/+*^; *Tie2*^*Cre+*^*;Vgll4*^*fl/-*^ and *Tie2*^*Cre+*^*;Vgll4*^*fl/-*^*;Yap*^*fl/+*^ embryos. VE-cad staining marks VECs (green). (D) Quantitative results of TUNEL+ cells of VECs and VICs in each individual leaflet (n = 5). White arrows point proliferating cells (in A) or apoptotic cells (in C). VEC: valve endothelial cell; VIC: valve interstitial cell. Scale bar = 100μm. *P<0.05, **P<0.01, ***P<0.005, ns: no significance.

As mentioned earlier, the Hippo pathway is critical for cell proliferation, in order to further confirm that the function of VGLL4 in valve remodeling is correlated with YAP, we examined cell proliferation using 5-ethynyl-2’-deoxyuridine (EdU) labelling and found that the proliferation of VIC and VEC in the leaflets of arterial valves in E15.5 *Tie2*^*Cre+*^*;Vgll4*^*fl/-*^ embryos increased dramatically compared with *Vgll4*^*fl/-*^*;Yap*^*fl/+*^ valves. However, we didn’t observe significantly increased proliferation in *Tie2*^*Cre+*^*;Vgll4*^*fl/-*^*;Yap*^*fl/+*^ valves (Fig 7A and 7B). These data implicate that VGLL4 not only play its role in development, but also in valve homeostasis at adult stage.

We then examined apoptosis at the same stages using TUNEL assay and found significantly reduced apoptosis of VIC in all the leaflets of arterial valves of *Tie2*^*Cre+*^*;Vgll4*^*fl/-*^ embryos. By contrast, we didn’t detect any significant changes in apoptosis of VIC in *Tie2*^*Cre+*^*;Vgll4*^*fl/-*^*;Yap*^*fl/+*^ arterial valves compare to *Vgll4*^*fl/-*^*;Yap*^*fl/+*^ controls (Fig 7C and 7D), indicating that semi-knockout of YAP rescued the programmed apoptosis of VIC in normal valve remodeling. Taken together, the above results demonstrate that proliferation and apoptosis are precisely regulated by Hippo signaling during semilunar valve development.

## Discussion

In this study, we used mouse genetics to determine the function of VGLL4 in mouse heart valve development. The results revealed an essential role of VGLL4 in heart valve morphogenesis via regulating VIC proliferation and apoptosis. In particular, we showed that deletion of VGLL4 resulted in progressive valve disease. Even as early as E15.5, *Vgll4*^*-/-*^ AoV and PV exhibited dramatically VIC over-proliferation. Further studies showed that loss of VGLL4 in endothelial cells resulted in abnormal arterial valves morphology and developed severe cardiac hypertrophy in 36-week-old (aged) mice, while semi-knockout of YAP rescued heart malformation induced by VGLL4 deletion. Altogether, our study shows that VGLL4 and precisely regulated Hippo signaling are required for proper heart valve remodeling and maturation.

The processes of valve development, remodeling and homeostasis are accompanied with cell proliferation, differentiation and apoptosis, which are precisely regulated by complex integration of multiple signaling pathways, such as Notch [20], Wingless-type MMTV integration site family (WNT) [21], Transforming Growth Factor-beta (TGFβ) [22], Bone Morphogenetic Protein (BMP) [23] and fibroblast growth factor (FGF) [24], vascular endothelial growth factor (VEGF) [25] pathways. The Hippo pathway plays important roles in cell differentiation, proliferation and apoptosis. YAP is a downstream effector of the Hippo pathway. However, the role of Hippo signaling in valve remodeling and morphology remains undisclosed.

Previously, we reported that YAP was required for EMT during heart cushion formation at E9.5. YAP knockout in endothelial cells reduced endocardial cell proliferation, led to hypocellular cardiac cushion and increased embryonic lethality [26]. In this study, we detected YAP remain widely expressed in post-EMT valves at E15.5, enlighten us that YAP also plays a role in post EMT valve development. VGLL4, a transcriptional co-repressor in the Hippo pathway, is critical for balancing cell proliferation and apoptosis during development and tissue homeostasis. As VGLL4 and YAP don’t contain DNA-binding domain and they mediate their biological functions mainly through interaction with TEAD transcription factor family [27]. Our previous studies have shown that VGLL4 could bind with TEAD4 through its TDU domains and directly competed with YAP for binding to TEAD4, which result in significantly suppressing cell growth in gastric and lung cancer cell lines with decreased expression of YAP/TEAD downstream genes including CTGF and CYR61, whereas deletion of both TDU domains attenuated the inhibitory function [14–16]. So we think during valve development, VGLL4 also exerts its inhibitory function through the competition for YAP-TEAD complex formation, as endothelial knockout of VGLL4 leads to endothelial origin VIC over-proliferation, further bolsters the inverse correlation between VGLL4 and proliferation capacity.

Based on our Vgll4-GFP reporter mice line we found that VGLL4 is extensively expressed in endothelial cells and valve region at E13.5, which enlightens us that VGLL4 plays its role in the post-EMT stage in which proliferation needs to be restricted. At the same time, apoptosis starts and valves begin to remodel and elongate into a thin valve. At E15.5, VGLL4 is expressed in all heart valves (AoV, PV, MV and TV), but restricted in AoV and PV and seldom detected in MV and TV at E17.5 (S10 Fig), this could explain our VGLL4 total knockout phenotype that MV and TV were normal on neonatal heart (S3 Fig). Moreover, from P0 to adult stage, VGLL4 is more inclined to locate in the fibrosa layer, the side in which the valve bears disturbed flow condition, where it is more likely for pathophysiologic changes such as sclerosis and calcification to occur, and where valve dysfunction, such as regurgitation and stenosis take place. The relationship between VGLL4 and fibrosa signaling in valve disease need to be further studied.

From our lineage trace data, we disclosed that VGLL4 mainly functioned in endothelial derived VICs, but not neuro crest derived VICs. Besides, we found the percentage of EdU^+^, RFP^-^ cells is not increased significantly in *Tie2*^*Cre*^*;Vgll4*^*fl/-*^*;Rosa*^*fsRFP/+*^ valves compared to *Tie2*^*Cre*^*;Vgll4*^*fl/+*^*;Rosa*^*fsRFP/+*^ controls (Fig 5C). This means that endothelial deletion of Vgll4 only affect endothelial and endothelial-derived VICs proliferation, but not RFP^-^ (non-endothelial-derived) VICs although they interacted with endothelial, these point enlighten us that Vgll4 function in endothelial derived VICs is cell autonomous effect.

VGLL4 total knockout lead to neonatal lethality, while endothelial knockout of VGLL4, heart cushion is normally formed and pups could live till adult stage, this is because in addition to the heart, VGLL4 also widely expressed in many tissues, such as bone, spleen, brain, uterus and so on, so we think the neonatal lethality in *Vgll4*^*-/-*^ is a comprehensive defect result, the function of VGLL4 in other organs need to be further investigated.

To our knowledge, this is the first *in vivo* genetic study showing the critical role of the Hippo pathway in regulation of valve remodeling and maturation. From lineage tracing data, we infer that endothelial but not neural crest VGLL4 regulates traced valve interstitial cell proliferation. This could be due to that VGLL4 is mainly expressed in endothelial cells and endothelial derived VIC. Thus, Hippo signaling in VEC is required for post-EMT semilunar valve morphogenesis and homeostasis. Precise regulation of the Hippo pathway is required for proper proliferation of endocardium derived mesenchymal cells and subsequent valve elongation. The present study identifies a previously unknown function of VGLL4 in valve remodeling and homeostasis. These findings suggest that mutations in VGLL4 may underlie human congenital heart valve dysplasia.

## Materials and methods

Ethics statement: The mice were housed in a specific pathogen-free environment at the Shanghai Institute of Biochemistry and Cell Biology (SIBCB) and treated in strict accordance with protocols approved by the Institutional Animal Care and Use Committee of Shanghai Institute of Biochemistry and Cell Biology (Approval number: SIBCB-S328-1511-052-C01)

### Generation of *Vgll4* mutant mice

We generated the *Vgll4* (NM_177683) targeted allele by knockout-first strategy, in which introducing a LacZ trapping element between exon1/2 and loxP sites flanking exon2 (ENSMUSE00000197361) through homologous recombination in ES cells from EUCOMM program (ID:78522) (S1A Fig). The presence of the 3’ arm (8.7kb) and 5’arm (8.6kb), targeted allele *Vgll4*^*LacZ*^ (413bp) and WT (470bp) alleles were confirmed by PCR genotype analysis (S1B–S1D Fig). Mice heterozygous for *Vgll4*^*LacZ/+*^ were bred to *Flp* trangenic mice, which deletes in the germline [28], resulting in *Vgll4*^*fl/+*^ mice which contain *Vgll4* flox allele (S1A Fig). A founder line (*Vgll4*^*LacZ/+*^) was crossed with female Sox2^*Cre+*^ [29] mice that express Cre in germline and produced total knockout allele *Vgll4* minus (*Vgll4*^*-*^) which contains LacZ trapping cassette (S1A Fig). Genotypes of *Vgll4* deletion band (-) (470bp) and conditional band (fl) (616bp) alleles were confirmed by PCR genotype analysis (S1E and S1F Fig).

### Generation of *Vgll4*^*Vgll4-eGFP/+*^ reporter mice

*Vgll4*^*Vgll4-eGFP/+*^ reporter mouse line carrying *Vgll4-eGFP-Wpre-polyA* expression cassette was knocked into the initial exon of the *Vgll4* gene via CRISPER/Cas9 strategy from Shanghai Biomodel Organisms Center, Inc. (S5A Fig). VGLL4-eGFP fusion protein expression is under the control of the endogenous VGLL4 promoter. The presence of the 3’ arm (2.4kb) and 5’arm (6.5kb) and targeted allele *Vgll4*^*Vgll4-eGFP*^ *allele* (749bp) and *WT* (405bp) alleles were confirmed by PCR genotype analysis (S5B–S5D Fig). The endothelium-specific *Tie2*^*Cre+*^ transgenic line [30], the neural crest-specific *Wnt1*^*Cre+*^ transgenic line [31] and *Rosa26*^*fsRFP*^ reporter [32] were acquired from the Jackson Laboratory. Embryos were collected from timed mating stage by daily monitoring for vaginal mucus plugs and the observation of the plug was considered E0.5. Primers used for genotyping were listed in S4 Table.

### Echocardiography

The left ventricle systolic function of mice at indicated stages (see main text) were assessed by echocardiography via a digital ultrasound system (Vevo2100 Imaging System, Visual Sonics). Conventional parameter of M-mode echocardiography measured were: heart rate (HR), interventricular septal thickness (IVS), left ventricular internal dimension (LVID), left ventricular posterior wall (LVPW), left ventricular volume (LV Vol), ejection fraction (EF), fractional shortening (FS) and left ventricular mass (LV Mass). All parameters were analyzed using original parameters and accompanying software. Pulse-wave Doppler analysis of the aorta was used to assess arterial insufficiency.

### Tissue preparation and histological analysis

Whole embryos (E12.5 or earlier), hearts (E13.5 to P10) and arterial roots (adult) were collected, fixed in 4% paraformaldehyde for 30 minutes and dehydrated with 30% sucrose overnight at 4°C. Tissues were embedded in Optimal Cutting Temperature (OCT) compound at -20°C for 30 min then stored in -80°C. Serial section were prepared at 10 μm thickness and collected on slides. Sirius Red/Fast Green staining was performed to determine collagen deposition as previously described [33]. Hematoxylin & Eosin staining performed according to standard procedures. The pathology of the heart valves was observed under Leica M165 FC stereo microscope or an Olympus BX53 microscope and the area of the heart valves were analyzed using Image-J software. We observed all sections and selected the largest valve section for further statistical analysis. The data were presented as mean ± SEM.

### EdU pulse-chase

For EdU labeling experiments, the EdU solution was dissolved in sterile 0.9% saline and injected subcutaneously in pregnant mice at the dose of 10 μg/g one day before collection. Embryos or hearts were collected and sectioned. EdU staining was then performed based on the Life Technologies Click-iT EdU Alexa 488 Imaging Kit protocol (Life Technologies, C10337).

### Immunostaining

Cryo-section slides were washed in PBS and fixed in 4% paraformaldehyde for 10 min. Slides were blocked in PBS containing 0.1% Triton X-100 and 5% normal donkey serum at room temperature for 1 h. After primary antibody incubation at 4°C overnight, signals were developed with Alexa Fluor secondary antibodies at room temperature for 30 min. Before mounting, tissues were counterstained with DAPI. Slides were examined by fluorescence microscopy (Olympus DP72) or laser confocal microscopy (Olympus FV-1200), as indicated. Antibodies used for immunostaining were listed in S5 Table.

### Western blot

Tissues were homogenized in Tris-SDS lysis buffer (50 mM Tris-HCl pH 8.0 and 1% SDS) and incubated on ice for 20 min, followed by centrifugation at maximum speed (22,000 g) to get the protein supernatant. All protein samples were mixed 1:4 with 4× loading buffer (10% SDS, 1.5 M dithiothreitol and 0.3 M Tris-HCl pH 6.8) and boiled for 5 min. Proteins were resolved by 10% SDS PAGE and transferred onto a polyvinylidene fluoride (PVDF) membrane (Immobilon, Millipore) using a Mini Trans Blot system (Bio-Rad). The membranes were then blocked in TBST (10 mM Tris-HCl pH 8.0, 150 mM NaCl and 0.5% vol/vol Tween-20) containing 5% skim milk powder at room temperature for 1 h. After that, membranes were incubated with the indicated primary antibody at 4°C overnight. The next day, membranes were washed and then incubated with HRP-conjugated secondary antibody at room temperature for 1 h. Signals were detected by enhanced chemiluminescence (Pierce) according to the manufacturer’s instructions. We generated VGLL4 antibody by ABclonal Biotechnology Co. Ltd. The detail information of antibodies used for western blot were listed in S5 Table.

### RNA extraction, reverse transcription, and Real-Time PCR

Aortic valves (AoV) and pulmonary valves (PV) were isolated from adult hearts. 4–5 valves from same genotype mice were pooled and treated as a single biological replication. Three such pooled samples were collected for each group for RNA isolation. RNAs were extracted from cells with TRIzol Reagent (Thermo Fisher) following standard procedures, and subjected to reverse transcription with ReverTra Ace qPCR RT Kit (TOYOBO). Real-time PCR were performed with SYBR Green Real time PCR Master Mix (TOYOBO). GAPDH served as internal control. A list of real-time PCR primers was included in S4 Table.

### Statistical analysis

Data for two groups were analyzed using an unpaired Student’s t-test, whereas comparison between more than two groups was performed using an ANOVA followed by Tukey’s multiple comparison test. Significance was accepted when P<0.05. All data are presented as mean ± SEM.

## Supporting information

S1 Data A package of the raw data for all the figures mentioned in our manuscript. (ZIP)

S1 Fig Generation of Vgll4-null and Vgll4-floxed allele. (A) The genomic structure of Vgll4 locus and targeting strategy. Coding exons are in yellow; noncoding exons are in grey, introns are shown using black solid lines, transcription initiation site is figured by a black arrow. FRT, Flp recombination target (green); LoxP, Cre recombination target (red). (B) Genotyping of 5' arm of WT (*Vgll4*^*+/+*^), heterozygous (*Vgll4*^*LacZ/+*^) and homozygous (*Vgll4*^*LacZ/LacZ*^) knockout first target allele. (C) Genotyping of 3' arm of WT, heterozygous and homozygous target allele. (D) Genotyping WT, heterozygous and homozygous of Vgll4 knockout first (LacZ) target allele. (E) Genotyping WT (*Vgll4*^*+/+*^), heterozygous (*Vgll4*^*+/-*^) and homozygous (*Vgll4*^*-/-*^) of Vgll4 minus allele. (F) Genotyping WT (*Vgll4*^*+/+*^) heterozygous (*Vgll4*^*fl/+*^) and homozygous (*Vgll4*^*fl/fl*^) of Vgll4 flox allele.
(XLSX)

S2 Fig Survival curve of Vgll4^-/-^ and Vgl4^+/-^, Vgll4^+/+^ control mice. (XLSX)

S3 Fig Histological analysis of neonatal heart valve area. (A) Neonatal heart sections from *Vgll4*^*-/-*^ and *Vgll4*^*+/-*,^ *Vgll4*^*+/+*^ control mice were performed HE staining that shows aortic valve (AoV), pulmonary valve (PV), mitral valve (MV) and tricuspid valve (TV). (B,C) Quantification of AoV area (B), PV area (C), from *Vgll4*^*-/-*^ (n = 12) and *Vgll4*^*+/-*^(n = 10), *Vgll4*^*+/+*^(n = 9) control mice. (D,E) Quantification of MV area (D) and TV area (E) from *Vgll4*^*-/-*^ (n = 6) and *Vgll4*^*+/-*^(n = 5), *Vgll4*^*+/+*^(n = 4) control mice. ***P<0.005, ns: no significance. Scale Bar = 200μm.
(XLSX)

S4 Fig VGLL4 null heart valves exhibit VICs overproliferation. Heart secitons from E15.5 *Vgll4*^*-/-*^ and *Vgll4*^*+/+*^ control mice and immunostaining via troponin I antibody (red) and proliferation marker EdU (green). Scale Bar = 200μm.
(TIF)

S5 Fig Generation of VGLL4-eGFP reporter mouse line. (A) The genomic structure of Vgll4 locus and targeting strategy. Coding exons are in yellow; noncoding exons are in gray, introns are shown using black solid lines, transcription initiation site is figured by a black arrow. Genotyping of the 5' arm (B) and 3' arm (C) of WT (*Vgll4*^*+/+*^), heterozygous (*Vgll4*^*Vgll4-eGFP/+*^) and homozygous (*Vgll4*^*Vgll4-eGFP/Vgll4-eGFP*^) target allele. (D) Genotyping WT, heterozygous and homozygous of target allele. WT: wild type; Mut: mutant.
(TIF)

S6 Fig Fate mapping of endothelial and neural crest lineages. Hearts from endothelial specific *Tie2*^*Cre+*^*;Rosa*^*fsRFP/+*^ (A) and neural crest specific *Wnt1*^*Cre+*^*;Rosa*^*fsRFP/+*^ (B) mouse line were obtained and sectioned, figures show AoV, PV, MV and TV region of RFP+ cell contribution from each lineage. AoV: aortic valve; PV: pulmonary valve; MV: mitral valve; TV: tricuspid valve. Scale Bar = 200μm.
(TIF)

S7 Fig Neonatal heart stained with endothelial marker VE-cad and DAPI. Scale bar: 200μm.
(XLSX)

S8 Fig VGLL4 negatively regulates VEC and VIC proliferation of arterial valves at adult stage. (A) EdU staining shows proliferating cells (green) in arterial valves of 8-week-old *Tie2*^*Cre+*^*;Vgll4*^*fl/-*^ and *Vgll4*^*fl/-*^ mice. POSTN staining marks VICs (red). (B,C) Quantitative results of EdU+ cells of VECs and VICs in each individual leaflet of AoV (B) or PV (C). (D) Quantitation of DAPI number per leaflet. (E) Quantitation of DAPI per mm^2^ valve area. (F,G) Immunostaining of ECM marker ColIII (F) and Versican (G). VEC: valve endothelial cell; VIC: valve intersitital cell; ECM: extracelluar matrix. n = 3. White arrow indicated proliferating cells. Scale bar = 100μm. ***P<0.005.
(TIF)

S9 Fig Immunostaining of YAP and VE-cad on E15.5 aortic valve. Scale bar:100μm.
(TIF)

S10 Fig Sections from E15.5 (A) and E17.5 (B) Vgll4-GFP mice were performed immunostaining of endothelial marker VE-cad in red and GFP in green. Mitral valve (MV), tricuspid valve (TV). Scale Bar = 100μm.
(TIF)

S1 Table Echocardiographic parameters of Vgll4^-/-^ and Vgl4^+/-^, Vgll4^+/+^ control mice. (XLSX)

S2 Table Echocardiographic parameters of *Tie2*^*Cre+*^*;Vgll4*^*fl/-*^ and *Vgll4*^*fl/-*^ control mice. (XLSX)

S3 Table Echocardiographic parameters of *Tie2*^*Cre+*^*;Vgll4*^*fl/-*^*;Yap*^*fl/+*^; *Tie2*^*Cre+*^*;Vgll4*^*fl/-*^; and *Vgll4*^*fl/-*^*;Yap*^*fl/+*^ mice. (XLSX)

S4 Table Primer list. (PDF)

S5 Table Antibody list. (PDF)
