# Supplementary figures and images for: VGLL4 plays a critical role in heart valve development and homeostasis
Source: PLoS Genet. 2019 Feb 21;15(2):e1007977. doi: 10.1371/journal.pgen.1007977 (PMC6400400; doi:10.1371/journal.pgen.1007977)

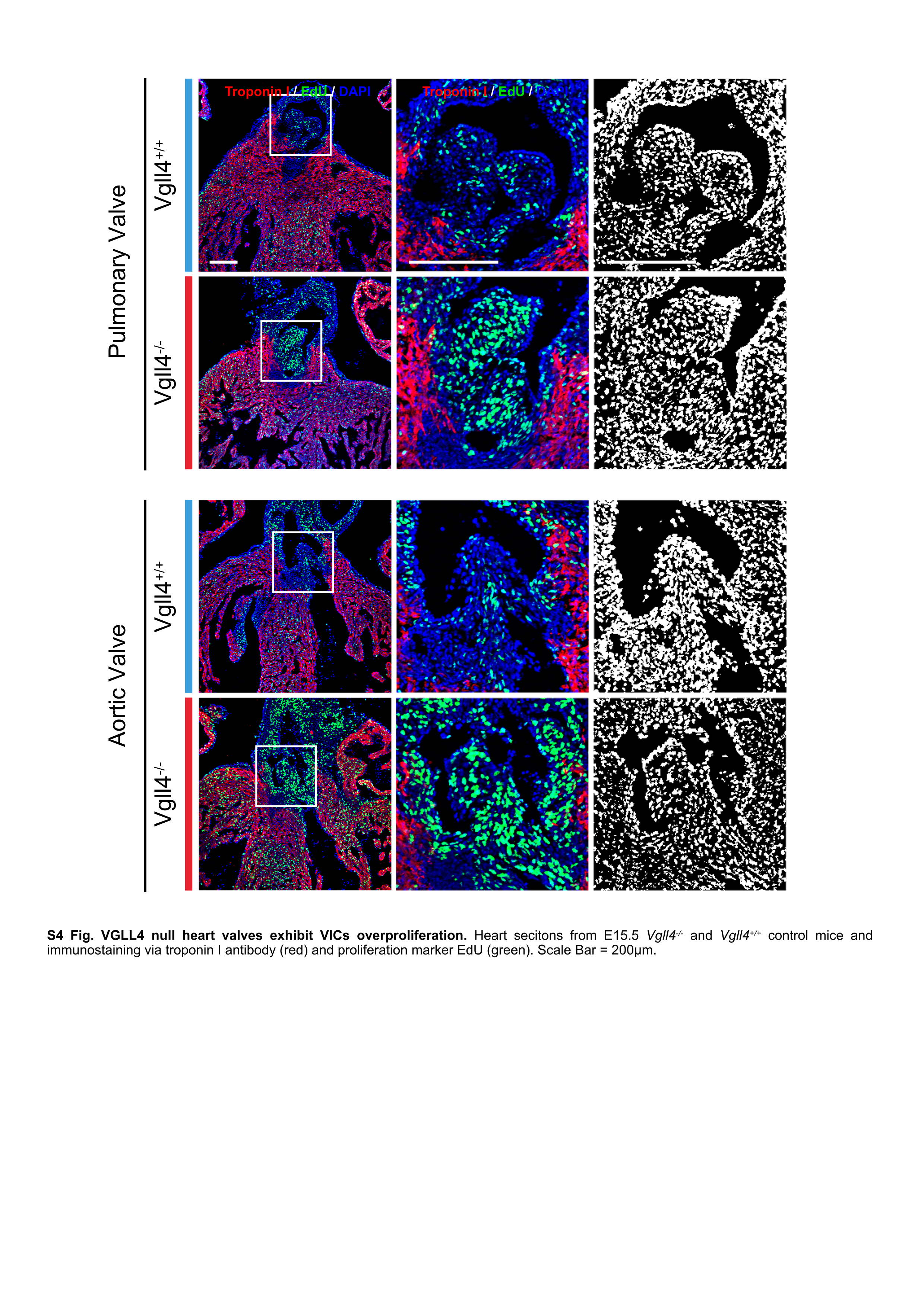

Supplement: S4 Fig — Heart secitons from E15.5 Vgll4-/- and Vgll4+/+ control mice and immunostaining via troponin I antibody (red) and proliferation marker EdU (green). Scale Bar = 200μm. (TIF) [file pgen.1007977.s005.tif]

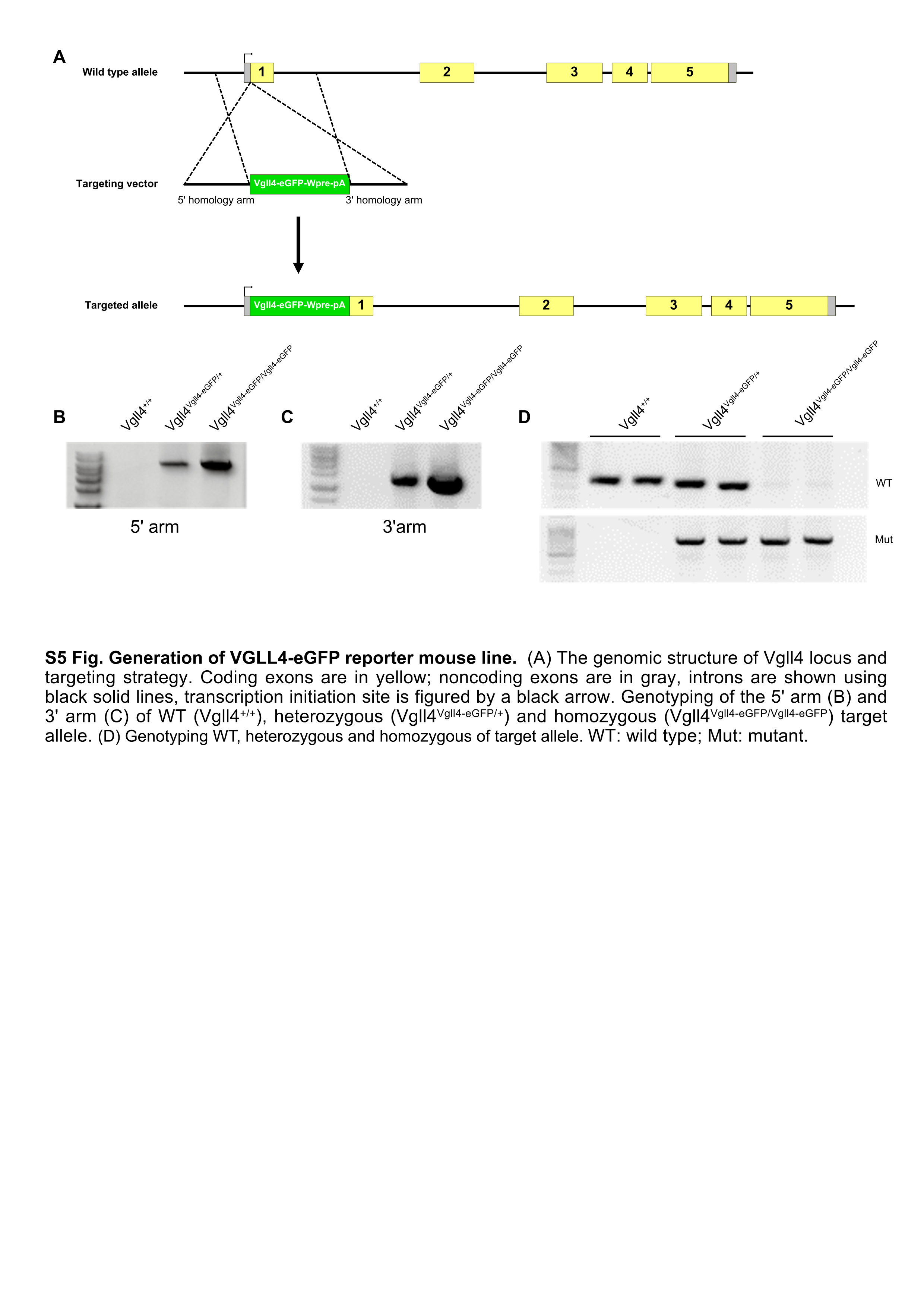

Supplement: S5 Fig — (A) The genomic structure of Vgll4 locus and targeting strategy. Coding exons are in yellow; noncoding exons are in gray, introns are shown using black solid lines, transcription initiation site is figured by a black arrow. Genotyping of the 5' arm (B) and 3' arm (C) of WT (Vgll4+/+), heterozygous (Vgll4Vgll4-eGFP/+) and homozygous (Vgll4Vgll4-eGFP/Vgll4-eGFP) target allele. (D) Genotyping WT, heterozygous and homozygous of target allele. WT: wild type; Mut: mutant. (TIF) [file pgen.1007977.s006.tif]

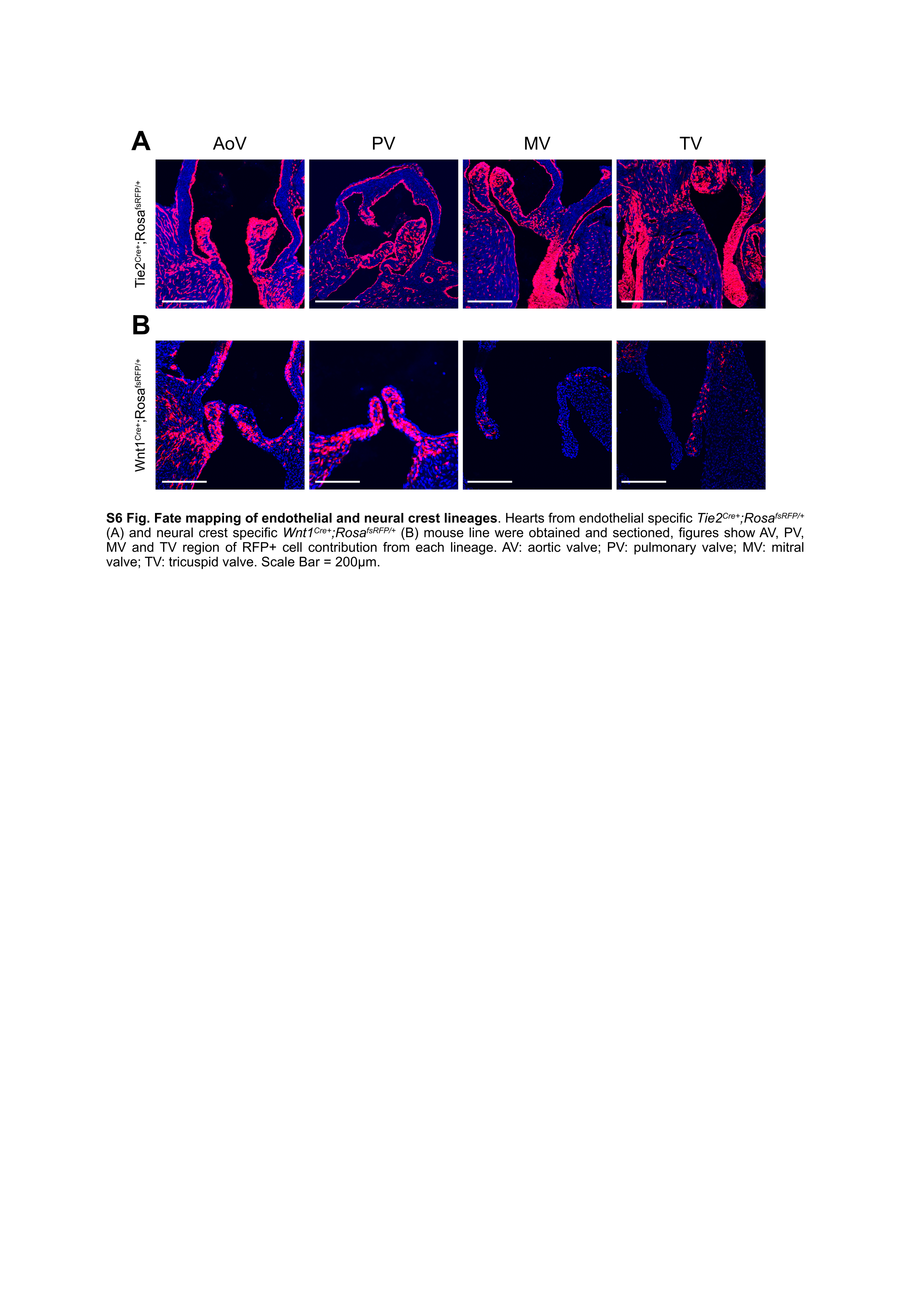

Supplement: S6 Fig — Hearts from endothelial specific Tie2Cre+;RosafsRFP/+ (A) and neural crest specific Wnt1Cre+;RosafsRFP/+ (B) mouse line were obtained and sectioned, figures show AoV, PV, MV and TV region of RFP+ cell contribution from each lineage. AoV: aortic valve; PV: pulmonary valve; MV: mitral valve; TV: tricuspid valve. Scale Bar = 200μm. (TIF) [file pgen.1007977.s007.tif]

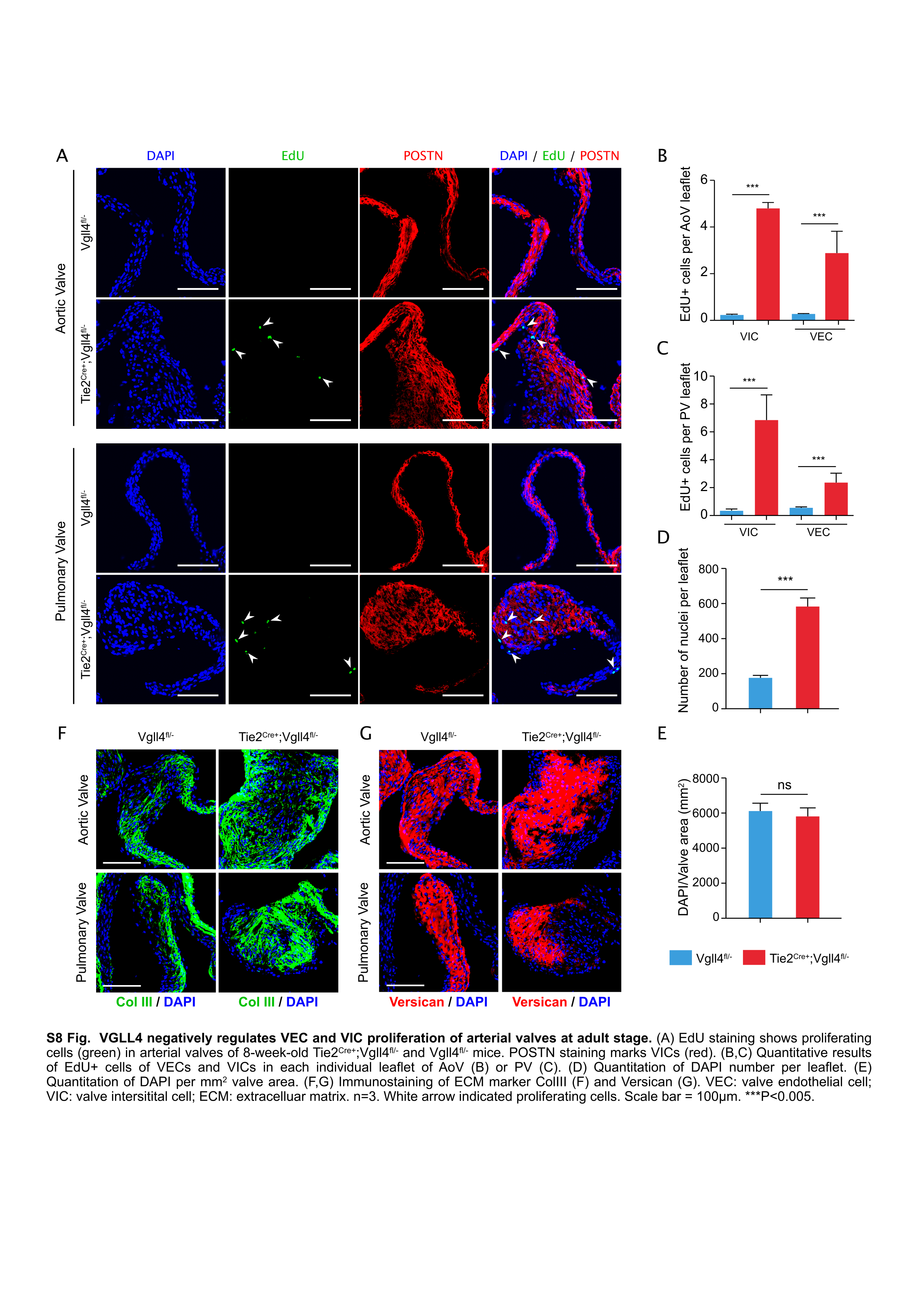

Supplement: S8 Fig — (A) EdU staining shows proliferating cells (green) in arterial valves of 8-week-old Tie2Cre+;Vgll4fl/- and Vgll4fl/- mice. POSTN staining marks VICs (red). (B,C) Quantitative results of EdU+ cells of VECs and VICs in each individual leaflet of AoV (B) or PV (C). (D) Quantitation of DAPI number per leaflet. (E) Quantitation of DAPI per mm2 valve area. (F,G) Immunostaining of ECM marker ColIII (F) and Versican (G). VEC: valve endothelial cell; VIC: valve intersitital cell; ECM: extracelluar matrix. n = 3. White arrow indicated proliferating cells. Scale bar = 100μm. ***P<0.005. (TIF) [file pgen.1007977.s009.tif]

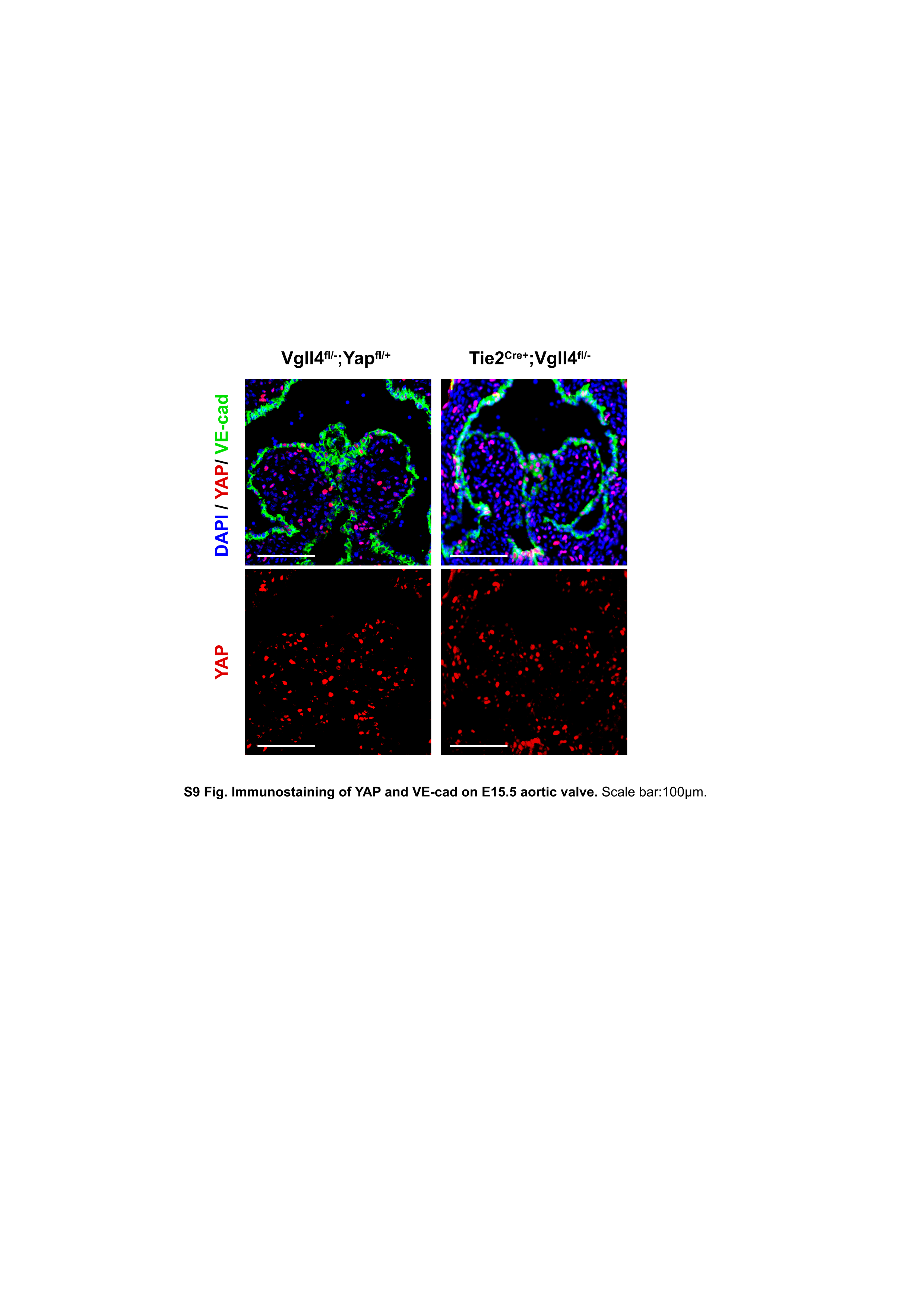

Supplement: S9 Fig — Scale bar:100μm. (TIF) [file pgen.1007977.s010.tif]

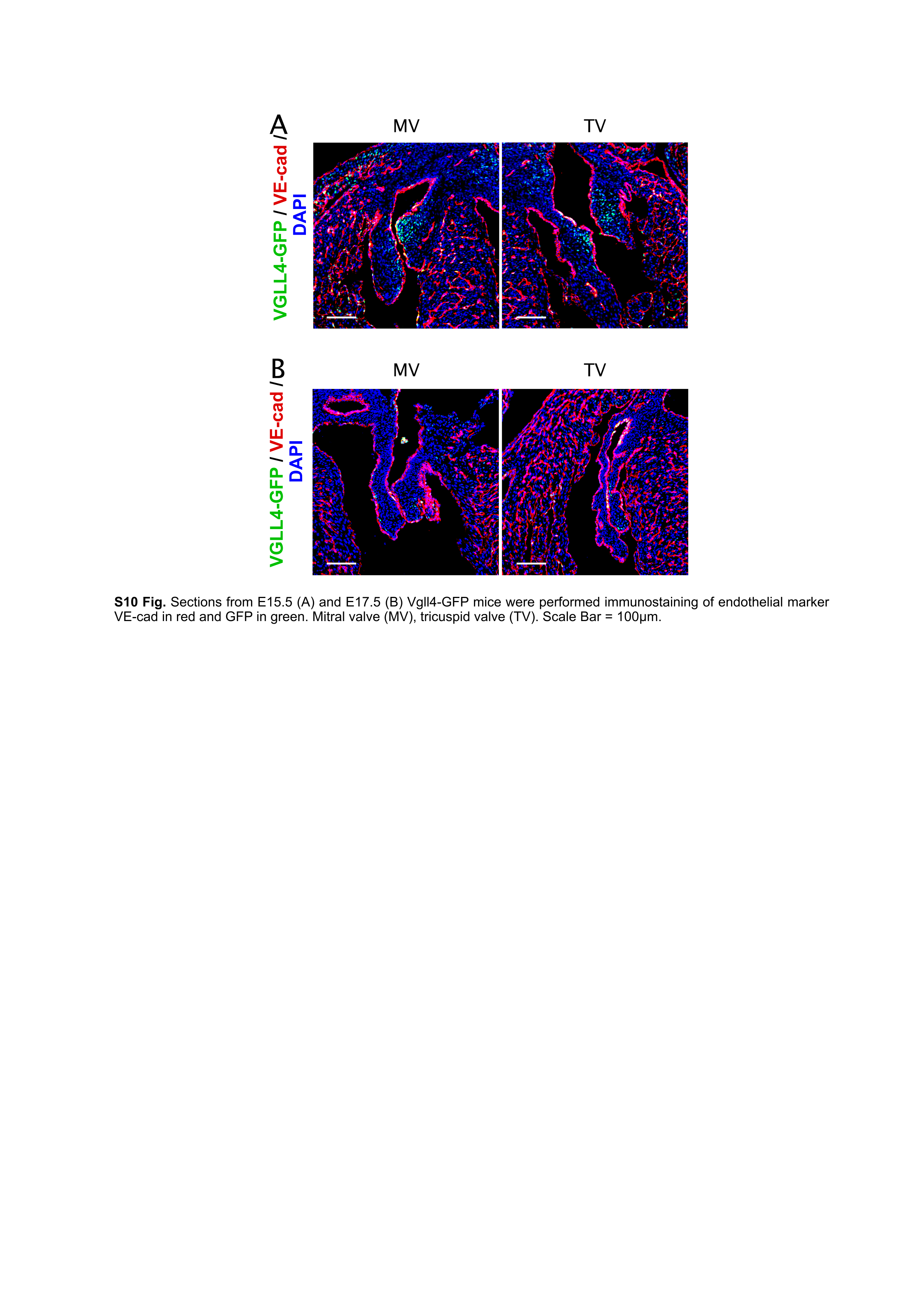

Supplement: S10 Fig — Sections from E15.5 (A) and E17.5 (B) Vgll4-GFP mice were performed immunostaining of endothelial marker VE-cad in red and GFP in green. Mitral valve (MV), tricuspid valve (TV). Scale Bar = 100μm. (TIF) [file pgen.1007977.s011.tif]
