# Supplementary material for: VGLL4 plays a critical role in heart valve development and homeostasis
Source: PLoS Genet. 2019 Feb 21;15(2):e1007977. doi: 10.1371/journal.pgen.1007977 (PMC6400400; doi:10.1371/journal.pgen.1007977)
Supplement: S4 Table — (PDF) [file pgen.1007977.s015.pdf]

S4 Table

Primer used for genotype

Gene	Forward	Reverse	Product size
Vgll4 LacZ	TTCCCGCCCGACTGCTGATAACAT	GGCTGCTCCCCTCCAACAT<br>ATTGCGTTGCGCCATCTCAGTCAG	WT:470bp; Mut:413bp
Vgll4 flox	TTCTTCTGGCCTCCATGGGCATTGT	ATGTTATCAGCAGTCGGGCGGGAA	WT:452bp; Mut:616bp
Vgll4 minus	TCCAGCTGAGCGCCGGTCGCTACCA	GACCACGTCTCCAAGATGAGCCGCA<br>GCATAATGGCCAGCAGAGGAGACTG	WT:350bp; Mut:470bp
Vgll4 eGFP	GCCTCTGTCTTCCTGAAGTT<br>GCCTAACTCGGTGTCCATC	TAACAGCGTGTCTCCTCCA<br>TGTTCTGCTGGTAGTGGTC	WT:405bp; Mut:749bp
Rosa RFP	AAGGGAGCTGCAGTGGAGTA<br>GGCATTAAAGCAGCGTATCC	CCGAAAATCTGTGGGAAGTC<br>CTGTTCCTGTACGGCATGG	WT:297bp; Mut:196bp

Primer used for quantitative RT-PCR

Gene	Forward	Reverse
Vgll4	ATGAACAACAATATCGGCGTTCT	GGGCTCCATGCTGAATTTCC
Yap	GCCATGCTTTCGCAACTGAA	CAAAACGAGGGTCCAGCCTT
Ctgf	TCCGGACACCTAAAATCGCC	TTCATGATCTCGCCATCGGG
Cyr61	AGAGGCTTCCTGTCTTTGGC	CTCGTGTGGAGATGCCAGTT
Survivin	AGAACAAAATTGCAAAGGAGACCA	GGCATGTCACTCAGGTCCAA
Cyclin A2	GGCTGACACTCTTTCCG	CTGGTAGCAAGAATTAGAGCAT
Cyclin B2	GAGAGTGAAGTCCTGGAA	GTGCTGATCTTCAGGAGT
Cyclin D1	TGAGAACAAGCAGACCATCC	TGAACTTCACATCTGTGGCA
Cdk4	GGGACATCAAGGTCACCCTA	CGCTTAGAAACTGACGCATT
Ki67	GCAGGTTAGCACTGTTATGAAAAC	GGGCCTTGGCTGTTTTACATT
Gapdh	GAAGGGCTCATGACCACAG	GATGCAGGGATGATGTTCTG
