# Supplementary material for: VGLL4 plays a critical role in heart valve development and homeostasis
Source: PLoS Genet. 2019 Feb 21;15(2):e1007977. doi: 10.1371/journal.pgen.1007977 (PMC6400400; doi:10.1371/journal.pgen.1007977)
Supplement: S5 Table — (PDF) [file pgen.1007977.s016.pdf]

S5 Table

Western Blot antibody

Antibody	Company	Catalog	Dilution
VGLL4	ABclonal		1:2000
β-Tubulin	DSHB	E7	1:5000

Immunostaining antibody

Antibody	Company	Catalog	Dilution
GFP	Abcam	ab6662	1:500
VE-cadherin	R&D	AF1002	1:500
WGA	Invirtrogen	W32464	1:500
RFP	Rockland	600-401-379	1:1000
Troponin I	Abcam	ab56357	1:200
WGA	Invirtrogen	W11261	1:500
Periostin	Abcam	ab14041	1:200
Versican	Millipore	ab1033	1:200
Collagen III	Southernbiotech	1330-01	1:500
YAP1	ABclonal	A1002	1:100
Alexa Fluor 555-conjugated donkey anti mouse IgG	Invirtrogen	A31570	1:1000
Alexa Fluor 488-conjugated donkey anti mouse IgG	Invirtrogen	A21202	1:1000
Alexa Fluor 555-conjugated donkey anti goat IgG	Invirtrogen	A21432	1:1000
Alexa Fluor 488-conjugated donkey anti goat IgG	Invirtrogen	A11055	1:1000
Alexa Fluor 647-conjugated donkey anti goat IgG	Invirtrogen	A21247	1:1000
Alexa Fluor 555-conjugated donkey anti rabbit IgG	Invirtrogen	A31572	1:1000
Alexa Fluor 488-conjugated donkey anti rabbit IgG	Invirtrogen	A21206	1:1000
